# Gene age shapes functional and evolutionary properties of the *Drosophila* seminal fluid proteome

**DOI:** 10.1073/pnas.2505490122

**Published:** 2025-10-07

**Authors:** Jose M. Ranz, Carolina Flacchi, Imtiyaz E. Hariyani, Alberto Civetta

**Affiliations:** ^a^Department of Ecology and Evolutionary Biology, University of California Irvine, Irvine, CA 92697; ^b^Department of Biology, University of Winnipeg, Winnipeg, MB R3B 2E9, Canada

**Keywords:** seminal fluid proteins, evolutionary gene age, interactome, *Drosophila* genus

## Abstract

Seminal fluid proteins (Sfps) are crucial for animal reproductive success, with Sfp-encoding genes believed to be evolutionarily young due to heightened rates of gene loss and gain and rapidly evolving at the sequence level relative to other genes. Using estimates of the phylogenetic origin of each *Drosophila melanogaster* Sfp gene, based on genomic resources that include outgroup species to the *Drosophila* genus, we examined the functional attributes and evolutionary characteristics of 357 Sfp genes relative to their evolutionary age. Contrary to the perception that many Sfp genes are evolutionarily young, 62% existed in the genome of the ancestor to this genus. These ancient genes have broader expression profiles, more expansive biological roles, and have more interactions with non-Sfp genes. This increased pleiotropy has imposed constraints on the rate of sequence evolution in ancient Sfp genes compared to younger ones. Notably, these younger Sfp genes evolve substantially faster as a result of both adaptive and nonadaptive evolutionary forces. Within the Sfp interactome, we identified a fast-evolving core subnetwork of younger genes with more restricted tissue expression and functions. Our systematic approach has uncovered a large set of ancient Sfp genes with distinct genomic, functional, and evolutionary characteristics compared to the younger, more commonly studied Sfp genes. With increased refined genomic and functional data acquisition across a wider variety of taxa, our approach and results serve as proof of the importance of systemic strategies applied to broadly defined gene sets based on their time of evolutionary origination.

## Significance

In many different taxa, seminal fluid proteins (Sfps) are transferred during copulation, eliciting in the female distinctive physiological and behavioral effects. Sfp-encoding genes have been regarded as a young gene category that features accelerated sequence differentiation. Our study shows that these rapidly evolving Sfp genes represent only a subset of the Sfp gene repertoire, with the majority having originated before the emergence of the genus *Drosophila*. Unlike ancient Sfp genes, younger ones show a narrower association with male reproductive function and reduced pleiotropy, forming a tight protein interaction subnetwork. Our findings emphasize the relevance of gene age in distinguishing between functionally expansive ancient Sfp genes and gene subnetworks, and a smaller group of younger ones that drive diversification and adaptation.

Seminal fluid proteins (Sfps) are key components of the male ejaculate that are transferred along with the sperm to the female during copulation. Sfps play central roles in postmating female processes, affecting individual fitness (1–4). In *Drosophila*, comparative genomic analyses have shown a dynamic Sfp gene complement, with many genes evolving rapidly in sequence and expression and experiencing high rates of duplication and loss (5–9). These evolutionary patterns have led to the view that Sfp-encoding genes experience rapid gene turnover, with limited detectable orthology even among closely related species (5–8, 10–13). However, the apparent absence of orthologs may partly reflect technical limitations, including overreliance on local alignment tools, variable genome assembly quality, inconsistent analytical pipelines across species and studies, and incomplete phylogenetic sampling. Consequently, no thorough analysis has clarified the origins and actual evolutionary age of the *Drosophila* Sfp gene complement.

These methodological limitations have hindered research into whether gene age affects the evolutionary and functional properties of Sfp genes (14, 15). As a result, differences in rates and mode of evolution may have been overlooked, along with whether ancient Sfp genes are primarily linked to reproduction. This gap in knowledge has two wider implications. The first is that the tendency to assign genes to broad functional categories can obscure important differences within groups that stem from the genes’ evolutionary age. Identifying these within-group differences can highlight specific genes or subgroups that act as drivers of diversification and adaptation, as well as genes whose functional impairment can be important for understanding variation in fitness and the etiology of deleterious phenotypes. The second implication is that not adopting a systematic approach prevents a full understanding of the evolution of the reproductive proteome function, one that recognizes polygenic phenotypic outputs, transitioning from single gene studies to systems biology (16).

Beyond individual gene characteristics, how Sfps have evolved as a network of interactive partners remains uncharacterized. Previous studies on the network topology and evolution of the *D. melanogaster* Sfp genes have been focused on the Sfp sex peptide (SP) due to its role in female postmating responses (17–19). SP binds to the sperm, which is facilitated by a network of at least eight additional Sfps (17, 19). The SP network genes appear to have originated prior to the emergence of one of the core SP functions—reducing female sexual receptivity after mating— with orthologs detectable as far back as species in the *Sophophora* subgenus (18, 20). However, an analysis of the entire Sfp proteome, considering the incorporation of new Sfp genes into the genome over evolutionary time, is lacking.

Here, we delineated a robust catalog of *D. melanogaster* Sfp-encoding genes based on high-confidence calls from two independent research groups (21–23), and characterized the functional and evolutionary properties of Sfp genes. We do so while exploring how these properties relate to the time of origin of these genes within the *Drosophila* lineage and more distant phylogenetic timepoints (15). This is possible thanks to a gene age categorization derived from upgraded genomic resources, increased phylogenetic sampling, and the combined use of sequence alignments and positional information that are insensitive to annotation quality (24). Additionally, we analyzed Sfps as an evolving protein network. Our findings offer significant insights into how Sfp genes have evolved in terms of their interactions and evolutionary age. Contrary to prior reports (5–8, 10), we find that a large portion of Sfp genes in *D. melanogaster* originated before the diversification of the genus *Drosophila*. These ancient Sfp genes are peripheral to the network, show pleiotropic expression and functionality, frequently interact outside the Sfp network, and exhibit slower nucleotide change rates. In contrast, younger Sfp genes dominate the core network, are enriched in reproductive functions, and show faster rates of change.

## Results and Discussion

### The Sfp Gene Complement Is Evolutionarily Ancient.

**Fig. 1.**
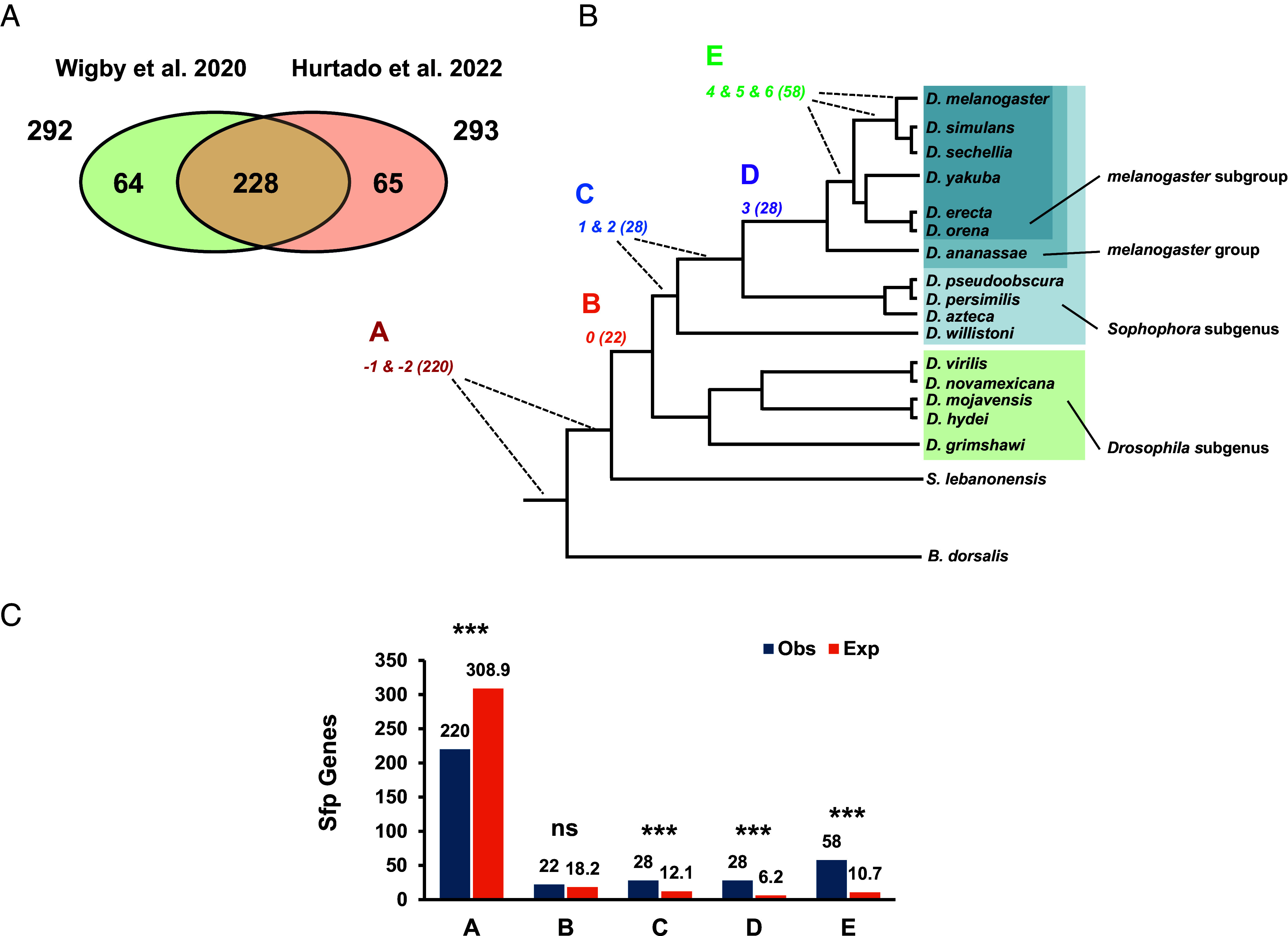
Age categorization of *D. melanogaster* Sfp-encoding genes. (*A*) Relationship of the datasets used to create a catalog of 357 high-confidence Sfp genes (21, 23). (*B*) Phylogenetic tree of the species used to infer the origin of Sfp-encoding genes (26, 27), with five gene age classes corresponding to the branch codes used by Dong et al. (15): class A, genes present before the *Drosophila* radiation (branches -1 and -2); class B, genes originated before the split between the *Drosophila* and *Sophophora* subgenera (branch 0); class C, genes formed in early divergent lineages leading to *D. willistoni* and *D. pseudoobscura* species groups (branches 1 and 2); class D, genes originated in the *D. melanogaster* species group (branch 3); and class E, genes present only in the *D. melanogaster* species subgroup (branches 4–6), including the *D. simulans* species complex and *D. melanogaster*. The number of Sfp genes originated within each age class is indicated in parentheses. (*C*) Clustered column plot showing the observed (blue) and expected (orange) counts of Sfp-encoding genes across age classes. Expected counts were calculated based on the proportion of these age classes in the entire *D. melanogaster* gene complement (15). The asterisks indicate particular age classes for which the difference between observed and expected counts was statistically significant according to post hoc tests to the chi-square test of independence and correcting for multiple tests: ns, nonsignificant; *, <0.05; **, <0.01; ***, <0.001 (28).

We cataloged 357 Sfp-encoding genes in *D. melanogaster,* combining results from two research groups using partially different criteria (Methods). Approximately two-thirds (228/357) of the Sfp gene candidates were common to both studies, with the rest equally contributed by each (Fig. 1*A* and Dataset S1). We then estimated each gene’s origin within the species phylogeny using gene ages inferred via a maximum parsimonious framework that leverages genome-wide pairwise alignments and assigns gene age based on both microsynteny and reciprocal best-hit support, involving 17 fly species plus *D. melanogaster* (15). This framework emphasizes both sequence conservation at the nucleotide level and positional conservation thus being less vulnerable to missing orthologs due to rapid sequence diversification, incomplete genome annotations, and genome assembly gaps. A parsimonious voting strategy across the phylogeny is used to infer presence of orthologs at ancestral nodes. Presence calls are further supported by the inclusion of a sizable subset of genome assemblies scaffolded with long PacBio HiFi reads, thus outperforming earlier calls, including some for the same species that utilized solely Sanger sequencing reads (25). The use of more contiguous genome assemblies lends further support to ortholog calls made across the species considered (15). Two species, *Scaptodrosophila lebanonensis* and *Bactrocera dorsalis*, from the Drosophilidae and Tephritidae families, respectively, serve as increasingly distant outgroups to the genus *Drosophila* (26, 27), providing essential phylogenetic depth for reliable gene age estimation.

All genes but one (LysC, a pseudogene) were categorized into one of five age classes spanning key sections of the species phylogeny (A-E from oldest to youngest; Fig. 1*B* and Dataset S1). We validated our gene age classification using independent data. Specifically, we used information from 90 *D. pseudoobscura* proteins, and 133 gene models from an upgraded annotation of *D. willistoni*, each with a 1-to-1 orthology relationship to *D. melanogaster* Sfp genes (29, 30). In total, we cross-checked 178 *D. melanogaster* Sfp genes (Dataset S1) that should belong to age classes A, B, or C, and found only five discrepancies (*Acp26Aa, Sfp33A1, Sfp53D, CG34051*, and *CG4271*), i.e. genes assigned to younger age classes (D and E) by Dong et al. (2025) (15). These discrepancies might result from errors in the gene age assignments, misidentifications in proteomic assays, or orthology call inaccuracies between *D. melanogaster* and *D. willistoni*. The fact that only 2.81% (5/178) of the checked Sfp genes showed discrepancies supports the reliability of the gene age classification adopted.

Two hundred and twenty Sfp genes originated prior to, and 136 during, the radiation of the *Drosophila* genus (class A vs classes B-E: 62.0% vs 38.0%; Fig. 1*B* and *SI Appendix*, Fig. S1*A*). However, age class A genes are underrepresented compared to their share in the entire genome (chi-square goodness-of-fit, χ^2^ = 333.74, d.f. = 4, *P* < 2.2 × 10^−16^) (Fig. 1*C* and *SI Appendix*, Table S1). This conclusion held when using the more conservative set of 228 Sfp genes (chi-square goodness-of-fit, χ^2^ = 333.71, d.f. = 4, *P* < 2.2 × 10^−16^) (*SI Appendix*, Fig. S1*A* and Table S2). Notably, these results show that Sfp genes are younger than most genes in the genome, but many (220/357) have an ancient origin that challenges the premise that Sfp genes have a relatively recent origin (5–8, 10). This pattern is exemplified by the nine known genes of the SP network (17, 31, 32). Of them, eight genes are part of age class A (*antr, aqrs, CG9997, CG17575, intr, lectin-46Ca, lectin-46Cb,* and *SP*), and one is part of age class B (*Sems*), meaning all were present prior to the diversification of the genus *Drosophila*. In fact, others have also found evidence of the presence of the gene *SP* in the genome of *S. lebanonensis* (33), in good agreement with our age categorization.

Our results are based on 1-to-1 orthology relationships allowing us to trace the origin of each *D. melanogaster* Sfp gene. Previous work that relied on comparative structural modeling and sequence homology searches, coupled with information on protein’s tissue of expression, pointed to functional similarities with mammalian proteins and a possible very ancient origin for some *Drosophila* SFPs (23, 34). We find a large proportion (78/98, or 80%) (Dataset S1) of those potentially very ancient Sfp genes present before the *Drosophila* radiation (i.e. part of age class A), raising the possibility that their evolutionary origin dates back to a common ancestor between protostomes and deuterostomes. Nevertheless, these possible cases of very ancient origin could also be explained by convergent evolution or paralogous relationships (23), and therefore, their true evolutionary age is not apparent at this time.

### Ancient Sfp Genes Are More Pleiotropic and Evolutionarily Constrained than Younger Sfp Genes.

Gene properties that denote higher pleiotropy such as high level and breadth of expression are thought to be correlated with gene age (14). Enhanced pleiotropy is thought to increase selective constraints, reducing evolutionary rates (35, 36). However, studies examining pleiotropy’s effect on selection efficacy (9, 37–39) have been inconsistent, partly due to conflicting results derived from interrogating different proxies of pleiotropy. We investigated whether different indicators of pleiotropy are evenly associated with the different age classes of Sfp genes.

**Fig. 2.**
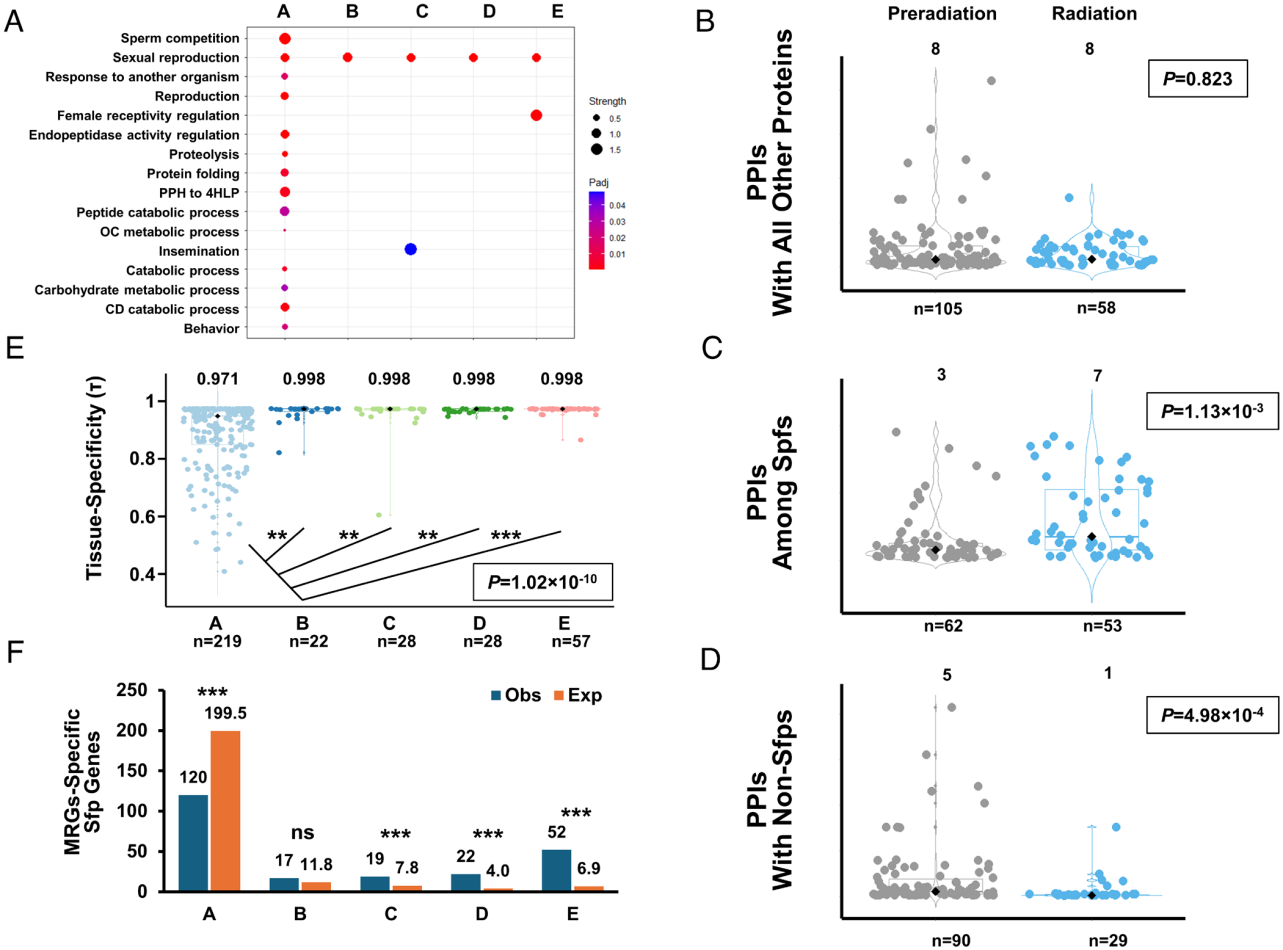
Ancient Sfps are more pleiotropic than younger Sfps. (*A*) Gene ontology enrichment based on FlyBase functional annotations followed by removal of redundant terms using REVIGO (41). The size of the dots in the dot plot is proportional to the gene-class enrichment, and the dots are colored based on FDR-corrected *P*-values. (*B*–*D*) Violin and box plots showing the distribution of the number of high-confidence protein–protein interactions (PPI) of the Sfps originated before and during the radiation of the *Drosophila* genus with other proteins, among Sfps, and with non-Sfps. (*E*) Violin (foreground) and box (background) plots showing the distribution of tau expression-specificity index, calculated using gene expression data from FlyAtlas2 (42), of Sfp-encoding genes of different age. Boxes represent the interquartile range (IQR) around the median (black diamond), and whiskers extend to 1.5 times the IQR. (*F*) Clustered column plot comparing the observed (blue) and expected (orange) number of Sfp-encoding genes showing male reproductive glands (MRGs) expression at τ≥0.9 across age classes. Expected counts were calculated based on the proportion of these age classes in the entire *D. melanogaster* gene complement (15). In violin plots, the *P-*value associated with the Kruskal–Wallis rank-sum test for differences across age classes is provided while the asterisks indicate the post hoc Wilcoxon rank-sum tests for which statistically significant differences were found after correcting for multiple tests (43). The median value for each distribution is shown on top. Statistically significant differences between observed and expected counts, according to post hoc tests to the chi-square test of independence and correcting for multiple tests, is indicated with asterisks: ns, nonsignificant; *, <0.05; **, <0.01; ***, <0.001 (28).

We evaluated whether age class A genes showed enhanced pleiotropy by assessing different attributes that can serve as proxies of pleiotropy. First, we examined each gene’s assigned Gene Ontology (GO) terms and found that only class A genes are enriched (5% FDR) beyond those strictly associated with reproduction (e.g. sexual reproduction, insemination, or regulation of female receptivity) (Fig. 2*A* and*SI Appendix*, Table S2). This conclusion did not change when we repeated this analysis comparing Sfp genes that originated before and after the radiation of the *Drosophila* genus (*SI Appendix*, Table S2). At this time, it is unclear whether this pattern is influenced by ancient orthologous genes being more extensively studied. Second, we retrieved protein–protein interaction (PPI) data for 163 Sfp genes (40) (Dataset S1). This includes interactions among Sfps and interactions with non-Sfps. One hundred and fifteen Sfp genes are predicted to interact with other Sfp genes, finding an equal representation of all age classes in the Sfp interactome (chi-square test of independence, χ^2^= 6.35, d.f.= 4, *P* = 0.174). Examining protein connectivity across age classes showed no significant differences (Kruskal–Wallis rank-sum test, χ^2^ = 5.06, d.f. = 4, *P* = 0.281) (*SI Appendix*, Fig. S2 and Table S3), which holds when comparing the more broadly defined age classes, *i.e.* before and during the *Drosophila* radiation (i.e. A vs B + C + D + E; Wilcoxon rank-sum test, χ^2^ = 0.05, d.f. = 1, *P* = 0.823) (Fig. 2*B*). However, when we excluded interactions with non-Sfps and restricted our analysis to interactions among Sfp proteins, we found significant differences (Kruskal–Wallis rank-sum test, χ^2^ = 14.255, d.f. = 4, *P* = 6.25 × 10^−3^), with age class A Sfps having significantly fewer interactions than the Sfps coded by genes in age class C, and a decreased number although not significant in relation to the Sfps from the age classes B and E (*SI Appendix*, Fig. S2 and Table S3). The comparison between the pre- and during radiation age classes substantiates even further the difference in their degree of connectivity within the Sfp interactome (Wilcoxon rank-sum test, χ^2^ = 10.6, d.f. = 1, *P* = 1.13 × 10^−3^) (Fig. 2*C*). Notably, the analysis of interactions outside the Sfp interactome revealed age class A Sfps with significantly more interactions than other age classes (Kruskal–Wallis rank-sum test, χ^2^ = 13.402, d.f. = 4, *P* = 9.5 × 10^3^) (*SI Appendix*, Fig. S2 and Table S3), which is again more obviously detected by the pre- vs during the radiation contrast (Wilcoxon rank-sum test, χ^2^ = 12.124, d.f. = 1, *P* = 4.98 × 10^−4^) (Fig. 2*D*). These results were consistent when considering only the 228 common Sfp genes (*SI Appendix*, Fig. S3).

Next, we investigated expression properties, hypothesizing that the broader functional range and interactive repertoire of class A Sfp genes would result in a broader expression profile. Indeed, class A genes have a significantly lower tissue-specificity (τ—tau index) compared to other age classes (Kruskal–Wallis rank-sum test, χ^2^ = 52.62, d.f. = 4, *P* = 1.02 × 10^−10^) (Fig. 2*E* and Dataset S1 and *SI Appendix*, Table S4), are less often male reproductive gland-specific (τ ≥ 0.9 and maximum expression in that tissue; chi-square goodness-of-fit, χ^2^ = 425.51, d.f. = 4, *P* < 2.2 × 10^−16^) (Fig. 2*F* and Dataset S1 and *SI Appendix,* Table S5), and are in general less likely to show tissue-specificity (τ ≥ 0.9 regardless of the tissue with maximum expression; chi-square goodness-of-fit, χ^2^ = 454.58, d.f. = 4, *P* < 2.2 × 10^−16^) (Dataset S1). These results are recapitulated when only the 228 common Sfp genes are considered (*SI Appendix*, Tables S4 and S5 and Fig. S4).

**Fig. 3.**
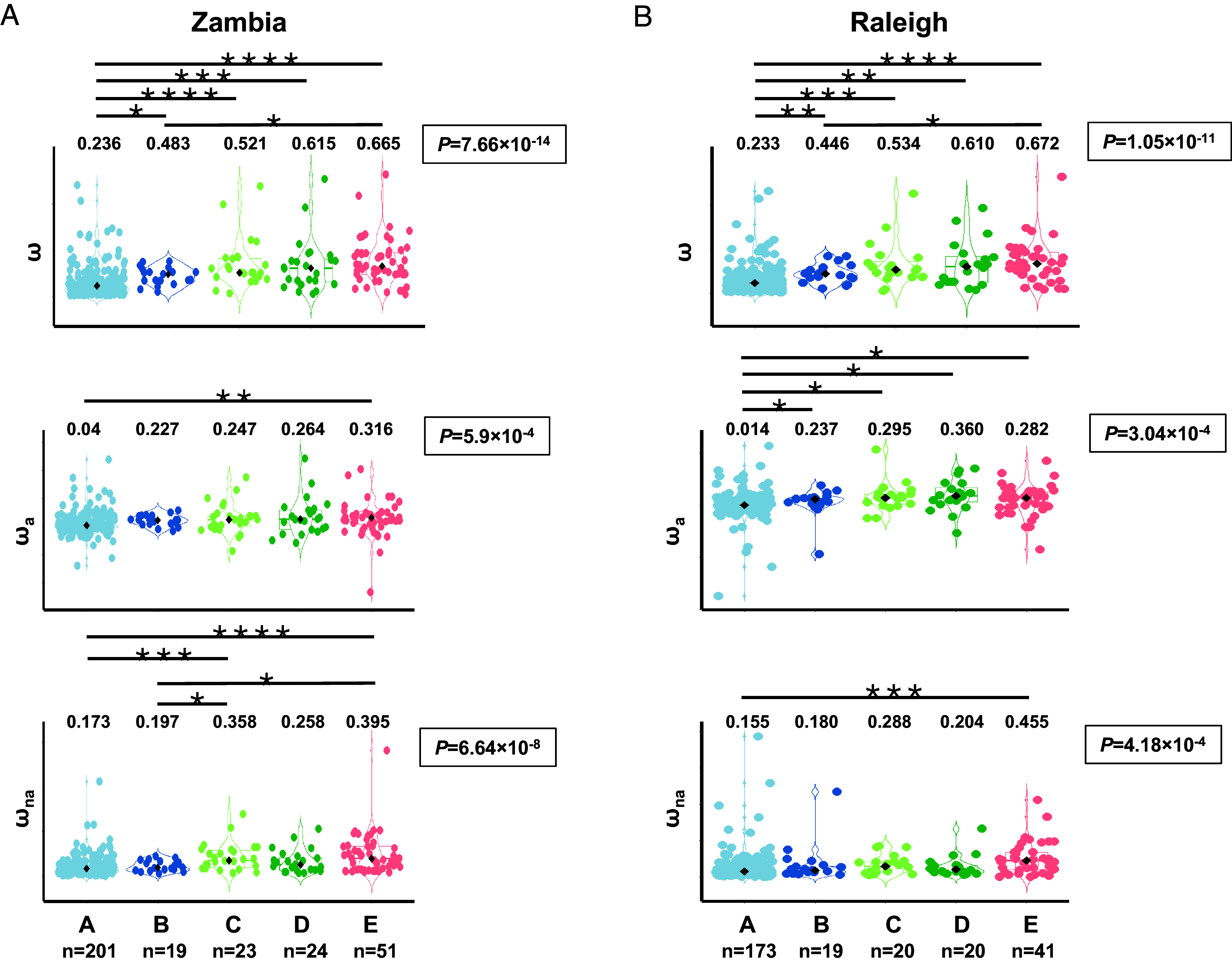
Distribution of coding sequence evolution metrics between *D. simulans* and two populations of *D. melanogaster* for Sfp genes across age classes. (*A*) The ratio of nonsynonymous to synonymous substitutions (ω), plus the adaptive (ω_a_) and nonadaptive (ω_na_) rates of evolution, between *D. melanogaster* and *D. simulans* as estimated using data from a *D. melanogaster* Zambia population. (*B*) Same estimates using data from a Raleigh population. Violin (foreground) and box (background) plots are provided for each age class by metric combination. Boxes represent the interquartile range (IQR) around the median (black diamond) and whiskers extend to 1.5 times the IQR. The median value for each distribution is shown on *Top*. The *P-*value associated with the Kruskal–Wallis rank-sum test for differences across age classes is provided on the *Right* of each contrast. Lines on *Top*, significant post hoc Wilcoxon rank-sum tests after correcting for multiple tests: *, <0.05; **, <0.01; ***, <0.001, ****, <0.001 (28).

Finally, we tested whether the increased pleiotropy of Sfp genes originated before the *Drosophila* radiation in terms of functional scope, protein connectivity, and expression breadth is associated with a slower rate of sequence coding evolution using inter- and intraspecific sequence information from the close relative *D. simulans* and two different *D. melanogaster* populations, one from Zambia (ZI) and one from Raleigh (RAL). The inclusion of these two populations allowed us to evaluate commonalities and differences between an ancestral (ZI) and a derived (RAL) population, thus calibrating the robustness of any possible finding. Sfp genes in age class A show lower ratios (ω) of nonsynonymous to synonymous divergence, i.e. they evolve slower than younger genes (RAL: chi-square goodness-of-fit, χ^2^ = 57.35, d.f. = 4, *P* = 1.1 × 10^−11^; ZI: chi-square goodness-of-fit, χ^2^ = 67.50, d.f. = 4, *P* = 7.7 × 10^−14^) (Fig. 3). Differences in the rate of evolution can be influenced by both adaptive and nonadaptive evolutionary mechanisms. To appraise the effect of these two mechanisms on the rate of evolution of Sfps in relation to age, we used the population polymorphism and divergence sequence data to estimate the proportion of substitutions fixed by positive selection (α) in order to derive estimates of adaptive (ωa) and nonadaptive (ωna) evolution, which provide a better understanding about how selection shapes gene evolution at the sequence level compared to using α alone (44, 45). We found that the lower evolutionary rate (ω) of ancient Sfp gene is a consequence of constraints in both their adaptive (ωa) and nonadaptive (ωna) evolutionary rates compared to Sfps of younger age classes (Fig. 3 and *SI Appendix*, Tables S6 and S7).

### The Sfp Interactome Includes Subnetworks with Distinct Functional and Evolutionary Properties.

**Fig. 4.**
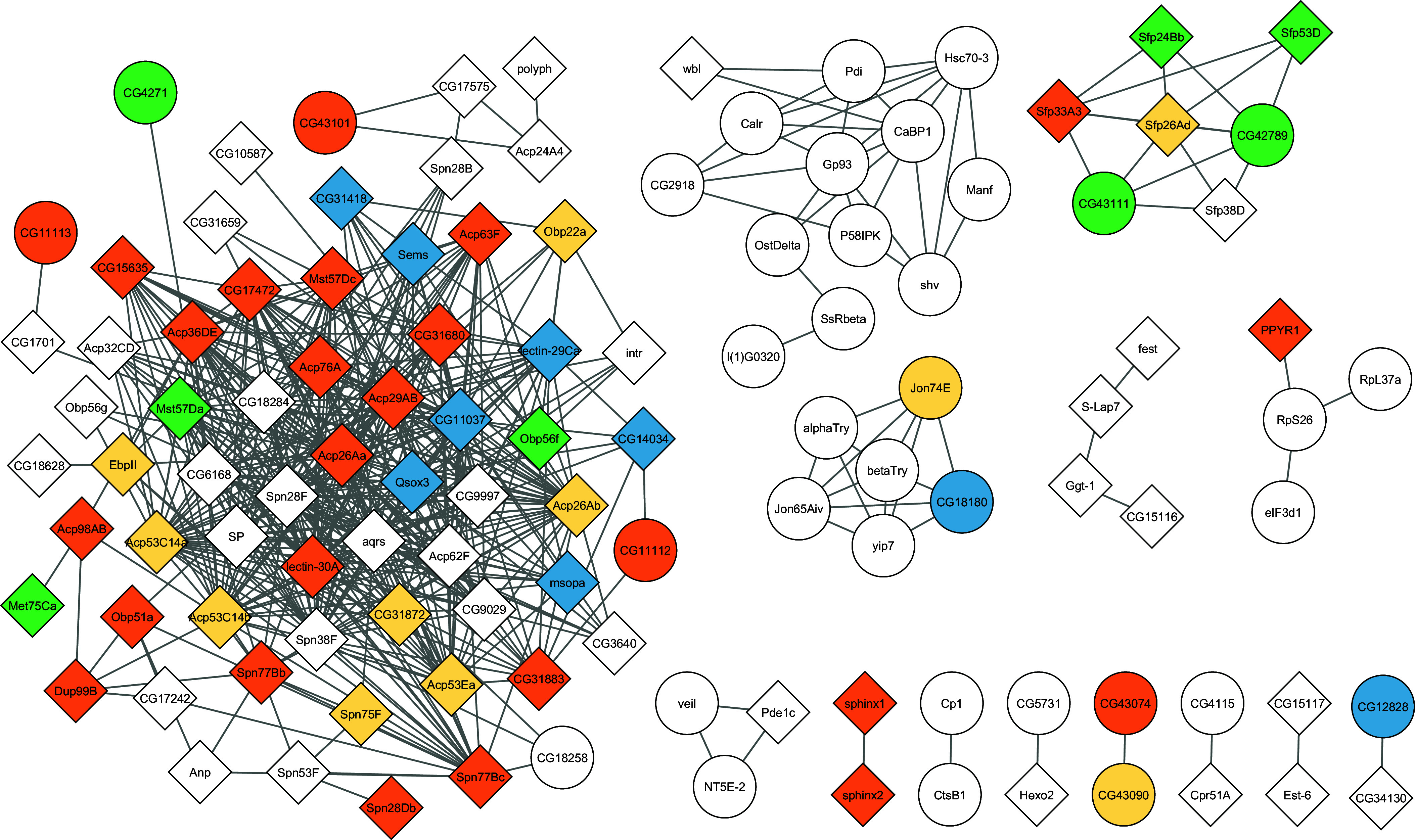
The Sfp protein–protein interaction network of *D. melanogaster*. The topology and composition of the Sfp interactome are shown. All 356 Sfp genes were considered. Only high-confidence interactions according to STRING (Methods) were included. Sfps with reproductive functions (i.e. those associated with the GO terms *sexual reproduction, reproduction, sperm storage, sperm competition, regulation of female receptivity, mating behavior,* and *insemination*) are indicated with diamonds, while nonreproductive Sfps are shown by circles. Age classes are color-coded: A (white), B (blue), C (yellow), D (green), and E (orange).

The differences in the number of PPIs between age class A and younger age classes, regarding interactions with both Sfps and unrelated proteins, raise questions about their localization and aggregation within the Sfp interactome. Among the 163 genes with available PPI information, we found 98 forming six subnetworks comprising ≥4 members, with one dominant, core subnetwork containing 56% (64/98) of Sfps (Fig. 4 and Dataset S1). The remaining 65 Sfps either did not form subnetworks above the minimum size or only interacted outside the Sfp interactome.

The core subnetwork expands as different age class Sfps are incorporated, being populated with many younger Sfps from the age class E (*SI Appendix*, Figs. S5 and S6). To quantify this and other trends, we assessed whether gene age is distributed evenly across subnetworks. The trend for enrichment for age class E Sfp genes was not statistically significant (20/64, *P*_adj_ = 0.1687, Monte Carlo simulations, n = 100,000) (*SI Appendix*, Table S8). This core subnetwork also includes all members, six, of the SP network with available PPI data (18). Further, the second- and third-largest subnetworks are significantly enriched for age class A and D Sfps, respectively (13/13, *P*_adj_ = 1.7 × 10^−4^, and 4/6, *P*_adj_ = 6.8 × 10^−3^, respectively). Importantly, in line with our GO term enrichment analysis, the genes in the core subnetwork are significantly associated with reproductive roles, while the second and fourth largest subnetworks are depleted of this type of genes (chi-square test of independence, χ^2^ = 60.22, *P*_adj_ = 5.0 × 10^−4^, 2,000 simulations; post hoc tests, *P*_adj_ < 1.0 × 10^−6^ for the three age classes) (*SI Appendix*, Table S9).

Sfps are known to be among the fastest evolving proteins at the sequence level between species (46–48). However, given the distinctive evolutionary dynamic of the core subnetwork, with younger genes enriched for reproduction functions and interactions with other Sfps, we predicted that Sfps in the core subnetwork would have different evolutionary rates relative to other Sfps. We found that the Sfp genes in the core subnetwork evolved faster (Mann–Whitney; Raleigh: Z = −2.95, *P* = 0.0031; Zambia: Z = −2.578, *P* = 0.0099), with population-specific differences in adaptive (ωa) and nonadaptive (ωna) evolution rates (*SI Appendix*, Fig. S7 and Table S10). In sum, these findings highlight unique functional and evolutionary characteristics of this relatively young reproductive subnetwork of the Sfp complement.

### Paralog Sfp Genes Have a Negligible Impact on Differential Age-Related Patterns.

A final consideration was the potential redundancy introduced by paralogous Sfp genes, which could affect the age-related differential patterns observed. To address this, we used DIOPT v9.0 (28) to identify paralog groups and reevaluated several key features of our analysis. In no case did treatment of data to account for repeated attributes within paralog groups altered our conclusions (*SI Appendix*). These results confirm that the distinct functional and evolutionary idiosyncrasies of the gene set are not confounded by paralog redundancy.

### Conclusions.

Our findings illuminate the age-dependent nature of the functional and evolutionary characteristics of Sfp genes. Contrary to the common belief that Sfp genes are rapidly evolving and young, we found a significant proportion of evolutionarily ancient Sfp genes. The broader functional repertoire and connectivity of ancient Sfp genes has imposed substantial constraints in their evolutionary dynamics. It is still uncertain whether evolutionarily ancient Sfps broadened their functional roles over time or were inherently diverse in their functions, being later repurposed as Sfps (8). Prior comparative structural modeling and enrichment analysis of protein classes identified *Drosophila* Sfps sharing structural similarities and functional characteristics with mammalian proteins, perhaps suggesting a later co-option of ancestral Sfps into species-specific reproductive-related functions (23, 34).

Our work illustrates the value of integrating gene age categorization into systematic analyses. Moving beyond broad functional categories allows us to detect patterns that emerge only when considering a gene’s evolutionary origin and its position within interaction networks. This perspective can reveal specific genes and gene networks with a disproportionate role in diversification and adaptation, providing leads for understanding the evolution of reproductive systems and the genetic basis of associated phenotypes.

Furthermore, our work relied on refined genomic and functional data from several *Drosophila* species and related Diptera lineages. As similar data become available for other vertebrate and invertebrate species, we will be able to determine whether the functional and evolutionary trajectories of comparable Sfp gene complements are similar in lineages with different interactome architectures, population parameters such as the effective population size, or mating systems with distinct intensities of selection. Finally, our identification of distinct subnetworks of Sfp interactions provides a resource that could be used to guide future biochemical and gene editing studies.

## Materials and Methods

### Sfp Gene Complement.

Two independent efforts have aimed to identify high-confidence Sfp-encoding candidate genes in *D. melanogaster* (21, 23). With different emphasis in the type of evidence used, these studies consider transcriptomic and proteomic data, sequence homology searches, computational predictions of the signal peptide, and phenotypic data derived from gene perturbation studies. Due to the difficulty in reliably delineating a high-confidence Sfp gene set, it is likely that both attempts are affected by false positives and negatives until systematic molecular and phenotypic studies are implemented for each candidate gene. Therefore, we focused on 357 Sfps gene candidates deemed as high confidence by either study and repeated key analyses using a more conservative set of 228 genes deemed likewise by both publications (Dataset S1).

### Phylogenetic Gene Age Dating.

Gene age inferences were based on syntenic alignments among 18 species of the *Drosophila* genus and two outgroup species, *S. lebanonensis* and *B. dorsalis* (15). Briefly, a parsimony framework implemented in the computational pipeline GageTracker (49) was used by Dong et al. (15) to assign genes to specific branches of the species phylogeny following Zhang et al. (50). Compared to previous attempts (25, 51), the new information used by the authors incorporated genomes from more species, including 10 new, and more contiguous genomes scaffolded with long PacBio HiFi reads. Beyond the *D. melanogaster* genome, all genome assemblies have a contig/scaffold N50 higher than 3 Mb. Further, two independent datasets of putatively present Sfp-encoding genes were included to validate the age inferences generated by Dong et al. 2025 (15) within the age classes A, B, and C. The first corresponds to a male accessory gland proteome characterization performed in *D. pseudoobscura*, which included explicit orthology calls in relation to *D. melanogaster* based on OrthoDB orthology assignments as implemented in FlyBase (29). Out of 163 predicted SFPs, 90 were present in our dataset. The second dataset corresponds to orthogroups established between the gene models of *D. willistoni* and *D. melanogaster* using Orthofinder (30, 52). Two hundred and ten Sfp-encoding genes in *D. melanogaster* have homologous relationship with orthogroups delineated in *D. willistoni*, 133 cases exhibiting a 1-to-1 orthologous relationship (Dataset S1).

### Sfp Interactions and Functional Properties.

RNAseq tissue expression values for 31 adult tissues and body parts from males and females were retrieved from FlyAtlas2 (42) and used to calculate the tissue specificity index of each Sfp gene (Tau Index, τ) (53). This index takes values from 0 to 1, with higher values denoting a narrower expression profile. We deemed a gene as tissue-specific when its index value was at least 0.9. The number of protein–protein interactions (PPI) for each Sfp and the construction of the Sfp network were predicted with STRING v12 (54) under the high confidence threshold and excluding text mining scores. The PPI network was visualized with Cytoscape v3.10 (55). Enrichment for biological process GO terms was examined within STRING at a 5% FDR (43) and redundant terms were excluded using REVIGO (41). Paralogous relationships among Sfp genes were determined based on information from DIOPT v9.0 (28).

### Rates of Nucleotide Sequence Evolution.

Synonymous and nonsynonymous nucleotide substitutions for each gene were retrieved from sequence comparisons between *D. simulans* and 197 lines from an African (Zambia, ZI) as well as 205 lines from a North American (Raleigh, RAL) population of *D. melanogaster* (56–58) using the iMKT R package (59). This package provides single-gene estimates for different population genetic parameters, relying in alignment pipelines and filtering criteria described elsewhere (57, 60), including divergence statistics derived from curated alignments between the two species (61). From iMKT, we downloaded the allele frequency spectrum and applied a 5% frequency threshold to estimate the number of neutral segregating sites in the nonsynonymous class (59). These corrected estimates were used to calculate α, the proportion of nucleotide substitutions driven by positive selection (59, 62), which was used in turn to calculate the rates of adaptive (ωa) and nonadaptive molecular evolution (ωna) (44, 45).

### Statistical Analyses.

Two-tailed Fisher’s exact test, chi-square goodness-of-fit, chi-square test of independence, analysis of residuals, Kruskal–Wallis rank-sum, Mann–Whitney pairwise tests, and the Benjamini–Hochberg correction for multiple testing were conducted using built-in functions in R (63). Monte Carlo simulations (n = 10,0000) were performed by resampling without replacement using an in-house script (64).

### Declaration of Generative AI and AI-Assisted Technologies in the Writing Process.

During the preparation of this manuscript, the authors used ZotGPT (GPT-4 Omni) to improve readability and language. After using this tool, the authors reviewed and edited the content as needed and take full responsibility for the content of the publication.

## Supplementary Material

Appendix 01 (PDF)

Dataset S01 (XLSX)

## Data Availability

All study data are included in the article and/or supporting information.
