# Supplementary material for: Gene age shapes functional and evolutionary properties of the Drosophila seminal fluid proteome
Source: Proc Natl Acad Sci U S A. 2025 Oct 7;122(41):e2505490122. doi: 10.1073/pnas.2505490122 (PMC12541329; doi:10.1073/pnas.2505490122)
Supplement: Supplementary file 1 — Appendix 01 (PDF) [file pnas.2505490122.sapp.pdf]

PNAS

Supporting Information for

Gene age shapes functional and evolutionary properties of the *Drosophila* seminal fluid proteome

Jose M. Ranz, Carolina Flacchi, Imtiyaz Hariyani, and Alberto Civetta

Jose M. Ranz and Alberto Civetta

Email: jranz@uci.edu; a.civetta@uwinnipeg.ca

This PDF file includes:

Supporting text

Figures S1-S6

Tables S1-S9

SI References

Other supporting materials for this manuscript include the following:

Dataset S1

Supporting Information Text

Impact of paralogous relationships on differences among age classes of Sfp genes

Paralogous Sfp-encoding genes, particularly those more recently integrated into the genome of *Drosophila* species, may exhibit similar functional and evolutionary properties potentially leading to redundancy (1, 2) that could be influencing our conclusions. To address this concern, we identified paralogous relationships among the Sfp genes of *D. melanogaster* using DIOPT v9.0 (3). Specifically, 159 Sfp genes identified as high or moderate ranked paralogs were included in downstream analyses (Dataset S1). These Sfp paralogous genes belong to 43 paralog groups. We then examined the heterogeneity of various features (see below) within paralog groups and tested how within-group homogeneity might account for deviations from the genome-wide age class observations. Finding no or very limited differences among the members of the same paralog group could be indicative of their similar functional or evolutionary properties and therefore they should not be treated and counted separately.

Gene age. A sizable percentage (44.2% or 19/43) of the paralog groups include paralogs that originated at distant branches of the species phylogeny, thus belonging to different age classes. The remaining 24 paralog groups contain paralogs that belong to the same age class: 20 associated with age class A; 1 with C; 1 with D; and 2 with E (including 55, 3, 3, and 5 paralogs, respectively, 66 in total). Considering these monoage paralog groups as single genes, or omitting all paralogs altogether, it still shows significant departures from the proportions of each age class when all genes in the genome are considered (*SI Appendix*, Table S1), i.e. age class A is underrepresented among the Sfp genes relative to the rest of the genome, but the Sfp gene complement as a whole is very ancient (*SI Appendix*, Table S1).

Gene Ontologies. To avoid arbitrary selection of representatives from monoage paralog groups, we evaluated the impact of paralogous Sfp genes by repeating the analysis of Sfp gene age classes enrichment for biological processes after omitting all paralogs. The results did not alter the patterns of enrichment previously identified when considering all Sfp genes (*SI Appendix*, Table S2).

Tissue of maximum expression. Based on data from FlyAtlas2 (4), 51% (22/43) of the paralog groups include paralogs that differ in which tissue they peak in expression. And for the remaining 21 paralog groups, i.e. those that are homogeneous for tissue of maximum expression, 19 exhibit maximum expression in male accessory glands, with 13 of them being simultaneously homogeneous for the age of their constituent paralogs: 8 belong to age class A; 1 to class C; and 1 to class E (including 23, 3, and 3 paralogs, respectively, 29 in total). The conclusion reached when all Sfp genes are treated independently remains unchanged when omitting all paralogs or when counting only one paralog per paralog group if the paralog group is also homogeneous for age class (*SI Appendix*, Table S5).

Rate of amino acid replacement. We calculated the coefficient of variation for the ω values across the 43 paralog groups and observed high levels of heterogeneity (mean±SD) within them: 66±45% in the Zambian population; and 62±33% in the Raleigh population. Subsequently, we tested if the coefficient of variation

differed between monoage and multiage paralog groups. We did not find differences in any of the two populations considered: Zambian population, Kruskal-Wallis rank sum test $X^2 = 0.52$, d.f. = 1, $P = 0.471$; Raleigh population, Kruskal-Wallis rank sum test $X^2 = 0.41$, d.f. = 1, $P = 0.523$). These results confirm that the magnitude of variation for ω among paralogs part of the same paralog group does not differ depending on whether the constituent members of the paralog group fall into the same or different age classes. Moreover, reanalysis of the ω estimates by removing all paralogs showed age class A Sfp genes with lower ω ratios in both the ZI and RAL populations, a consequence of constraints in adaptive (ω_a) and non-adaptive (ω_{na}) evolution. These results are consistent with those obtained when using the complete set of Sfp genes (*SI Appendix*, Table S6).

Protein-protein interaction networks. We examined the extent to which paralogs from the same paralog group are present in the same subnetwork of protein-protein interactions. Forty paralogs from 14 paralog groups are part of one of the six subnetworks shown in Fig. 4. Subnetworks 2, 3, 5 and 6 either harbor zero or only one Sfp paralog gene from the same paralog group while the other two subnetworks harbor two or more Sfp paralog genes from the same paralog group. These are subnetwork 1, with 42 (out of 64 total genes in the subnetwork, being part of 12 different paralog groups), and subnetwork 4, with all 6 Sfp genes in this subnetwork being paralogs (from 2 different paralog groups). If we add the condition that the Sfp paralog genes must belong to the same age class, we find 25 paralogs from 10 paralog groups as part of the indicated two subnetworks. These 25 paralogs fall across the different age classes in the following way: 16 in A; 2 in C, and 7 in E. Testing for enrichment of particular age classes across the six delineated subnetworks by having monoage paralog groups in the same subnetwork represented by one single paralog, or by omitting all paralogs altogether, did not alter any of the conclusions reached in the main text when considering all Sfp genes as part of the mentioned subnetworks (Monte Carlo simulations, n = 100,000; *SI Appendix*, Table S8).

Overall, there is a sizable number of cases of paralog groups showing internal patterns of heterogeneity for one or more of the features analyzed, which justifies treating Sfp paralog genes separately as opposed to jointly. These findings are in fact expected as 74.8% of the Sfp paralog genes belong to age classes A and B, meaning that they were present before or at the origin of the genus *Drosophila*, i.e. predating the radiation of the subgenera *Drosophila* and *Sophophora*. Such ancient paralogs have had a fair amount of time to diversify for different functional and evolutionary features and attributes, reducing their degree of redundancy. In conclusion, considering Sfp paralog genes from the same paralog group separately has negligible effects on the analyses conducted in the main text.

Supporting Figures

• Fig. S1. Age categorization of *D. melanogaster* Sfp-encoding genes common to two previous publications.

• Fig. S2. Differences in PPIs among Sfps of different age classes.

• Fig. S3. Violin and box plots showing the distribution of the number of PPIs established by Sfps encoded by genes of different age classes.

• Fig. S4. Clustered column plot comparing the observed and expected number of Sfp-encoding genes featuring expression specificity across age classes.

• Fig. S5. Stepwise formation of the Sfp protein-protein interaction network of *D. melanogaster* for the 356 Sfp-encoding gene candidates.

• Fig. S6. Stepwise formation of the Sfp protein-protein interaction network of *D. melanogaster* for the 228 Sfp-encoding gene candidates common to two studies.

• Fig. S7. Rate of evolution of core subnetwork Sfp genes vs other Sfp genes.

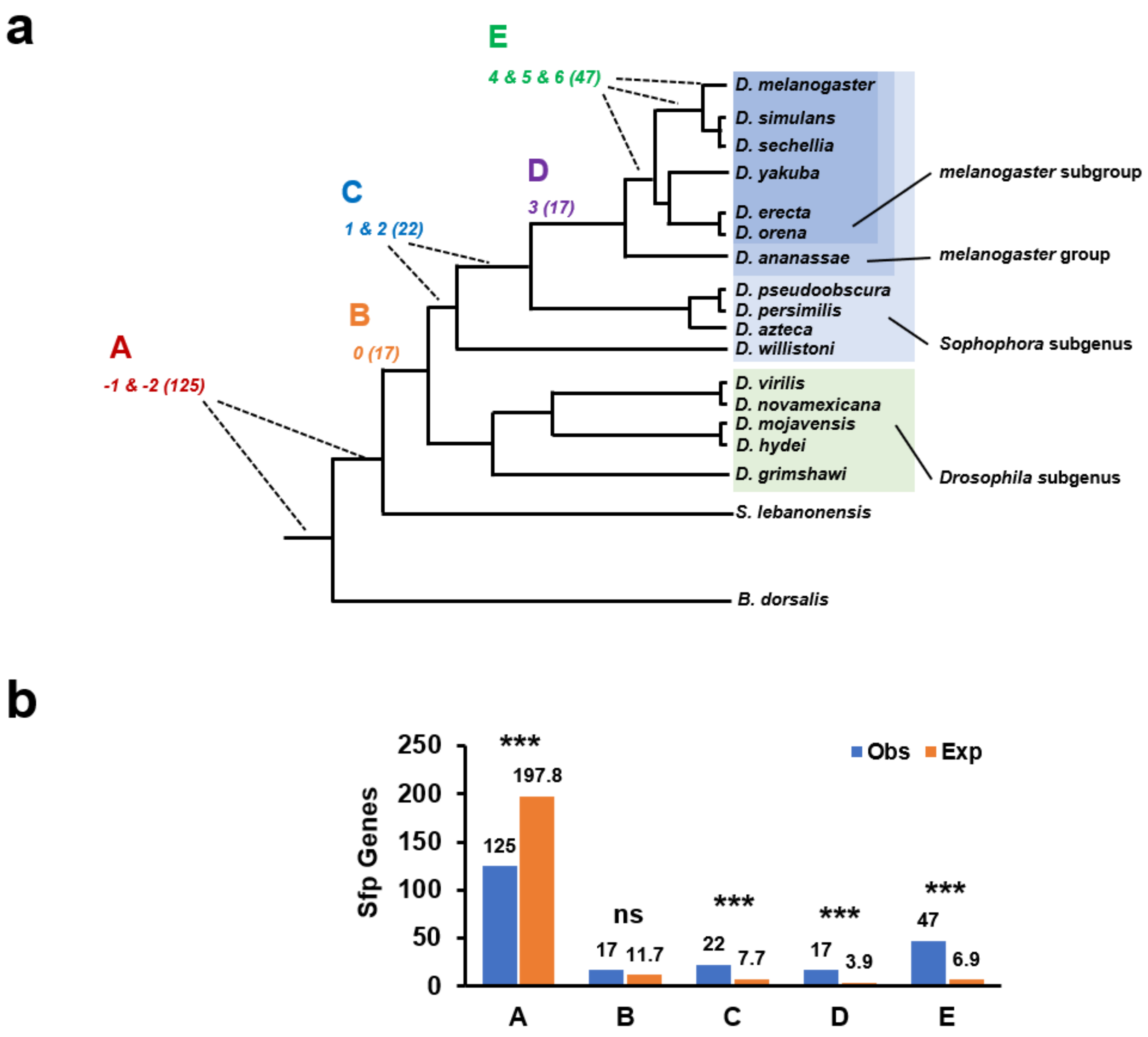

Figure S1. Age categorization of *D. melanogaster* Sfp-encoding genes common to two previous publications.

a Phylogenetic tree of the species used to infer the origin of Sfp-encoding genes (5, 6), with five gene age classes corresponding to the branch codes used by Dong et al. (7): class A, genes present before the *Drosophila* radiation (branches -1 and -2); class B, genes originated before the split between the *Drosophila* and *Sophophora* subgenera (branch 0); class C, genes formed in early divergent lineages leading to *D. willistoni* and *D. pseudoobscura* species groups (branches 1 and 2); class D, genes originated in the melanogaster species group (branch 3); and class E, genes present only in the *D. melanogaster* species subgroup (branches 4-6), including the simulans species complex and *D. melanogaster*. The number of Sfp genes originated within each age class is indicated in parentheses. Only the consensus set of 228 Sfp genes were considered. **b** Clustered column plot showing the observed (blue) and expected (orange) counts of Sfp-encoding genes across age classes. Expected counts were calculated based on the proportion of these age classes in the entire *D. melanogaster* gene complement (7). The asterisks indicate particular age classes for which the difference between observed and expected counts are statistically significant according to post hoc tests to the chi-square test of independence and correcting for multiple tests: ns, nonsignificant; *, <0.05; **, <0.01; ***, <0.001 (8).

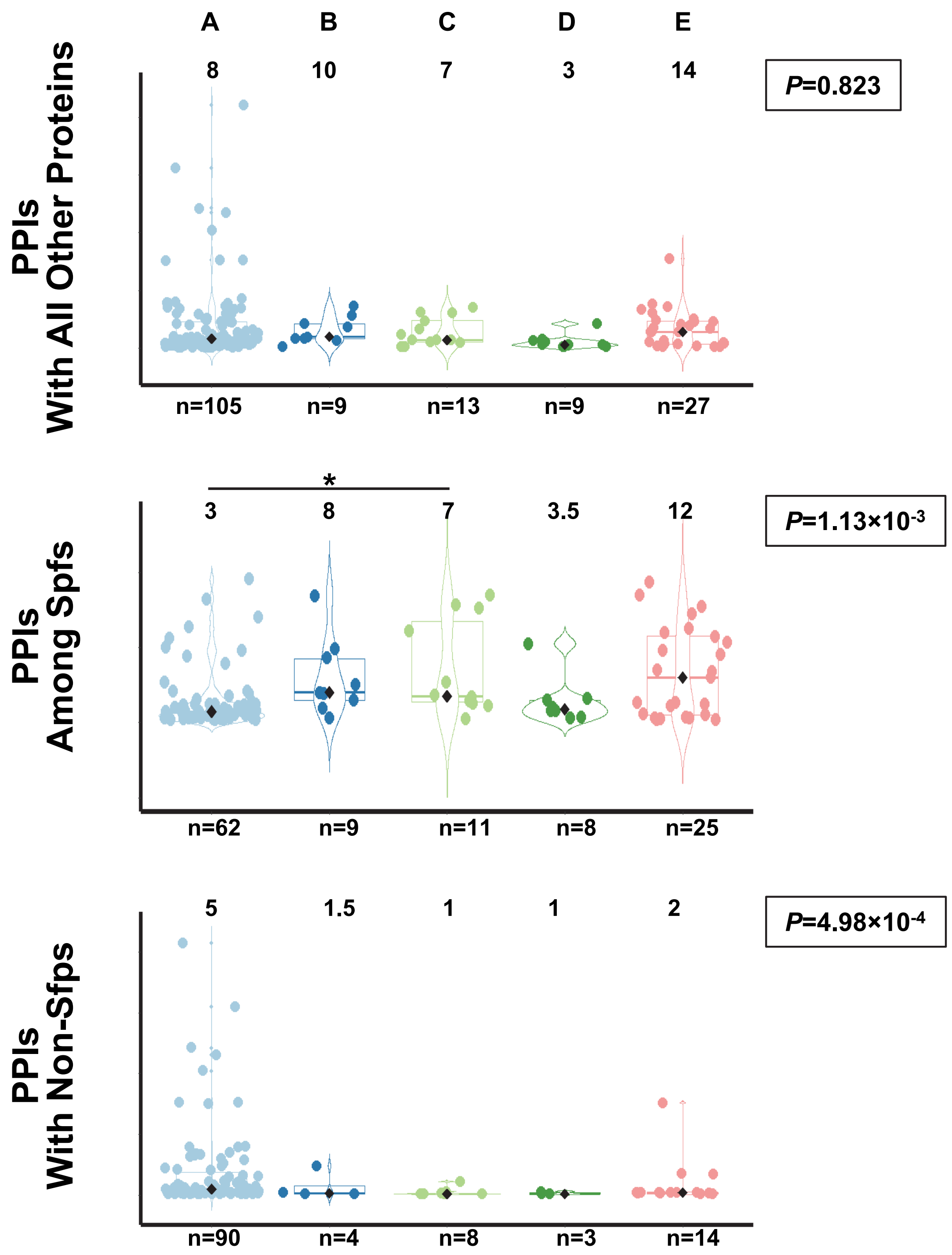

Figure S2. Differences in PPIs among Sfps of different age classes.

Violin (foreground) and box (background) plots showing the distribution of the number of high-confidence protein-protein interactions (PPI) of Sfps whose encoding genes originated at different age classes (A-E). From top to bottom, all interactions, among Sfps, and with non-Sfps. Only the consensus set of 228 Sfp genes were considered. Boxes represent the interquartile range (IQR) around the median (black diamond) and whiskers extend to 1.5 times the IQR. The median value is shown on top. The *p*-value associated with the Kruskal-Wallis rank sum test for differences across age classes is provided on the right of each contrast. Lines on top, significant post hoc Wilcoxon rank-sum tests after correcting for multiple tests: *, <0.05; **, <0.01; ***, <0.001, ****, <0.001 (8). The outcome of the post hoc tests can be found in *SI Appendix,* Table S4.

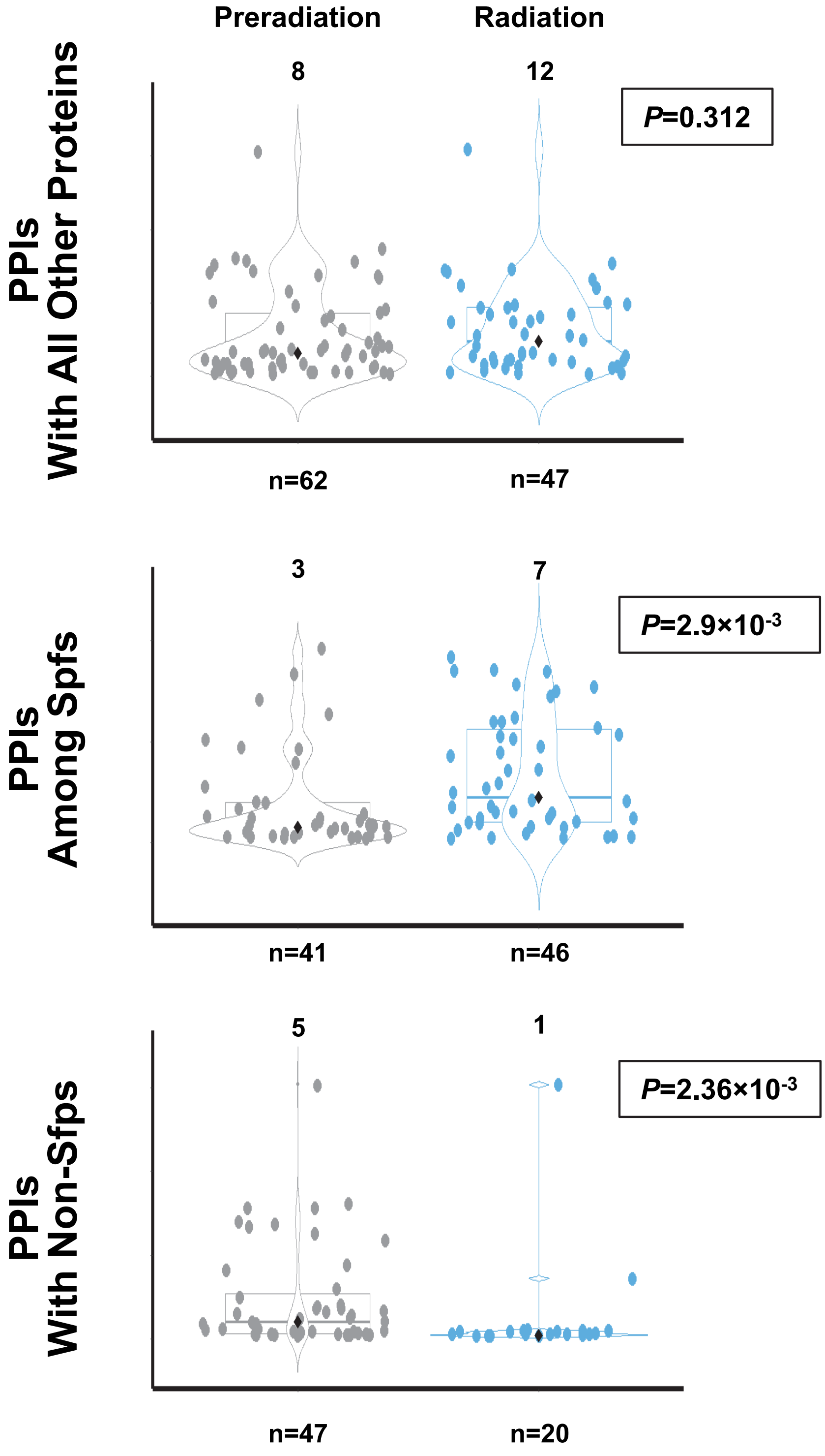

Figure S3. The distribution of the number of PPIs established by ancient vs. younger Sfps.

Violin and box plots are shown in the foreground and background, respectively. Only the consensus set of 228 Sfp genes were considered. Boxes represent the interquartile range (IQR) around the median (black diamond) and whiskers extend to 1.5 times the IQR. The median value for each distribution is shown on top. The broadly defined pre-radiation (ancient) and radiation (young) age categories correspond to the A and the rest of age classes (*i.e.* B+C+D+E) combined. The *p*-value associated with the Wilcoxon rank sum test with continuity correction is provided on the right of each contrast.

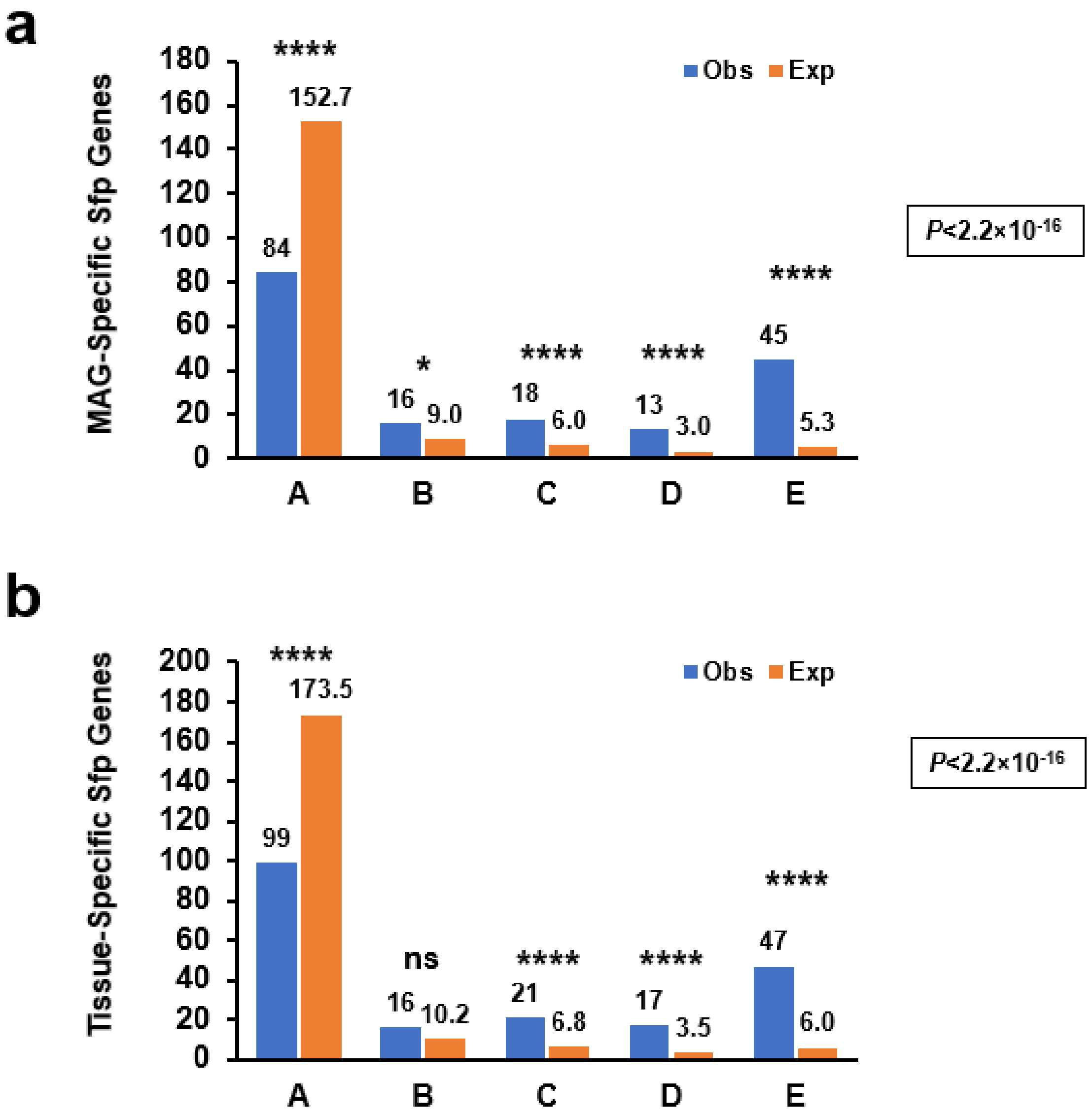

Figure S4. Clustered column plot comparing the observed and expected number of Sfp-encoding genes featuring expression specificity across age classes.

Observed (blue) and expected (orange) number of Sfp-encoding genes showing (**a**) male reproductive gland expression (MAG), and (**b**) tissue-specific expression in general. Tissue expression specificity is associated with $\tau \geq 0.9$. Only the consensus set of 228 Sfp genes were considered. Expected counts were calculated based on the proportion of these age classes in the entire *D. melanogaster* gene complement (7). The *P* values resulting from the corresponding chi-square goodness-of-fit tests are shown. Tau values were calculated using information from FlyAtlas2 (4). Statistically significant differences between observed and expected counts, according to post hoc tests to the chi-square test of independence and correcting for multiple tests, is indicated with asterisks: ns, nonsignificant; *, <0.05; **, <0.01; ***, <0.001.

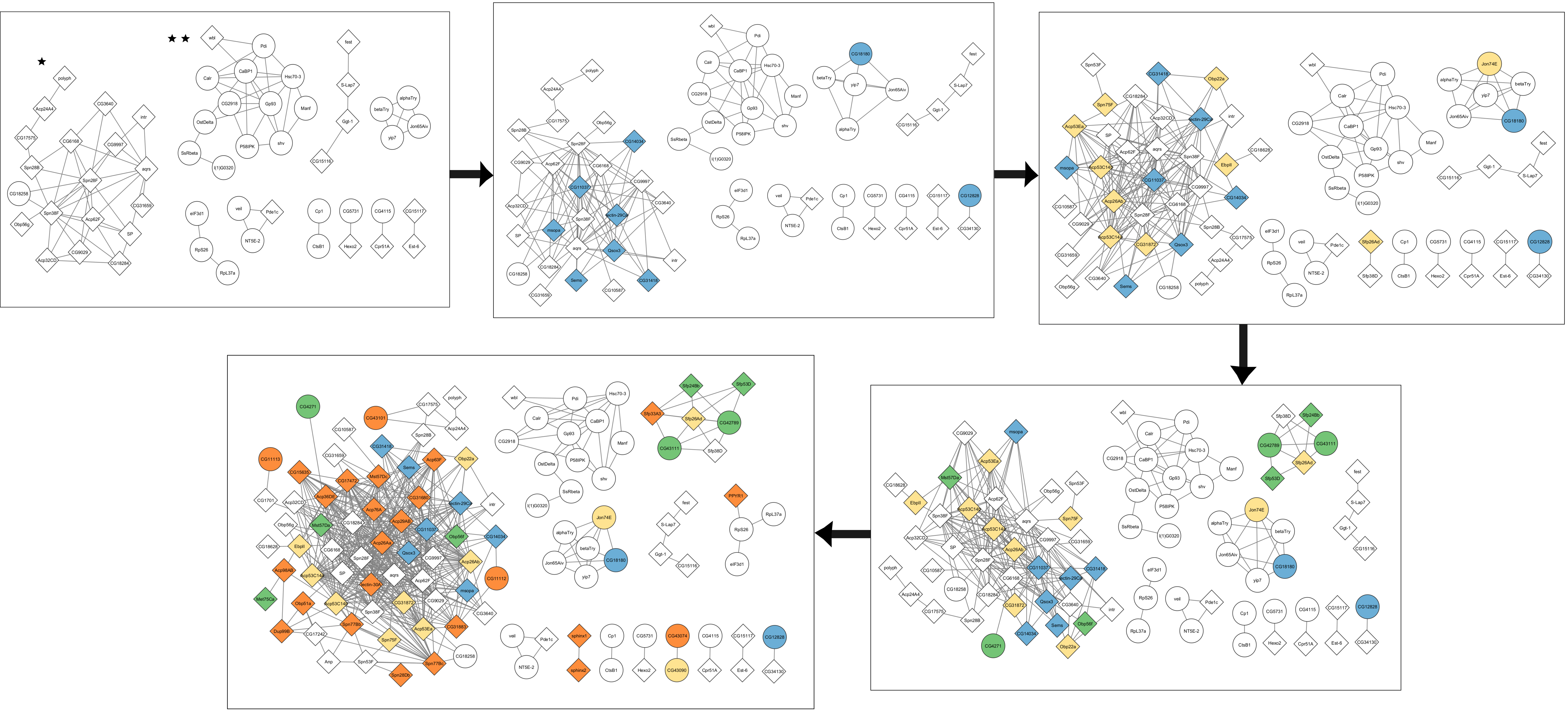

Figure S5. Stepwise formation of the Sfp protein-protein interaction network of *D. melanogaster* for the 356 Sfp-encoding genes.
The topology and composition of the Sfp interactome are shown clockwise as proteins from progressively younger age classes are added, starting from the top left (only age class A) and ending at the bottom left (all age classes combined). The 356 Sfp-encoding genes dubbed as high-confidence in at least one of two studies, Hurtado et al. 2022 (9) and Wigby et al. 2020 (10), were considered. Only high-confidence interactions according to STRING (Methods) were considered. Sfps with reproductive functions (i.e. those associated with the GO terms sexual reproduction, reproduction, sperm storage, sperm competition, regulation of female receptivity, mating behavior, insemination) are indicated with diamonds, while non-reproductive Sfps are shown by circles. Age classes are color-coded: A (white), B (blue), C (yellow), D (green), and E (orange). The core and second-largest subnetworks are indicated with one and two stars, respectively.

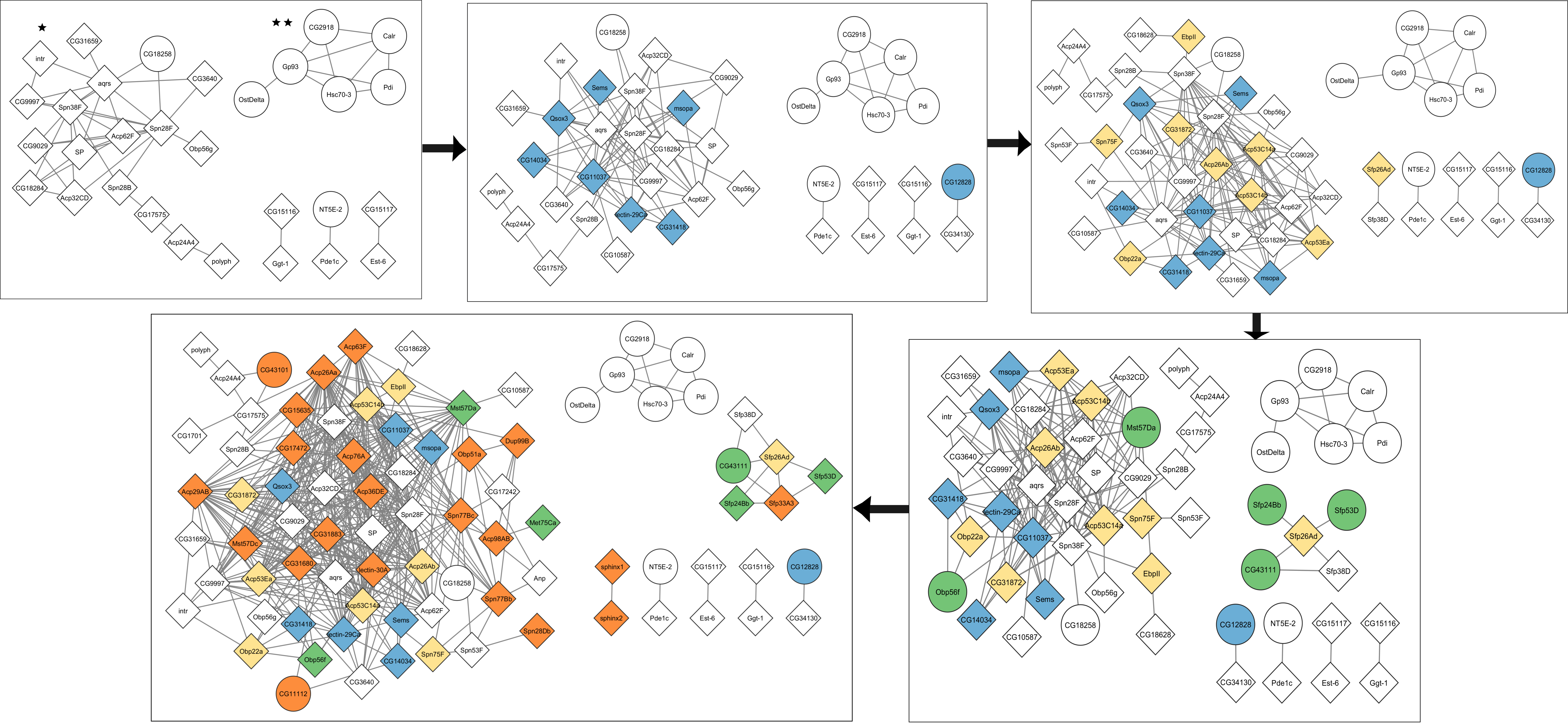

Figure S6. Stepwise formation of the Sfp protein-protein interaction network of *D. melanogaster* for the 228 Sfp-encoding genes common to two studies.

The topology and composition of the Sfp interactome are shown clockwise as proteins from progressively younger age classes are added, starting from the top left (only age class A) and ending at the bottom left (all age classes combined). The 228 Sfp-encoding genes dubbed as high-confidence in two studies, Hurtado et al. 2022 (9) and Wigby et al. 2020 (10), were included. Only high-confidence interactions according to STRING (Methods) were considered. Sfps with reproductive functions (i.e. those associated with the GO terms sexual reproduction, reproduction, sperm storage, sperm competition, regulation of female receptivity, mating behavior, insemination) are indicated with diamonds, while non-reproductive Sfps are shown by circles. Age classes are color-coded: A (white), B (blue), C (yellow), D (green), and E (orange). The core and second-largest subnetworks are indicated with one and two stars, respectively.

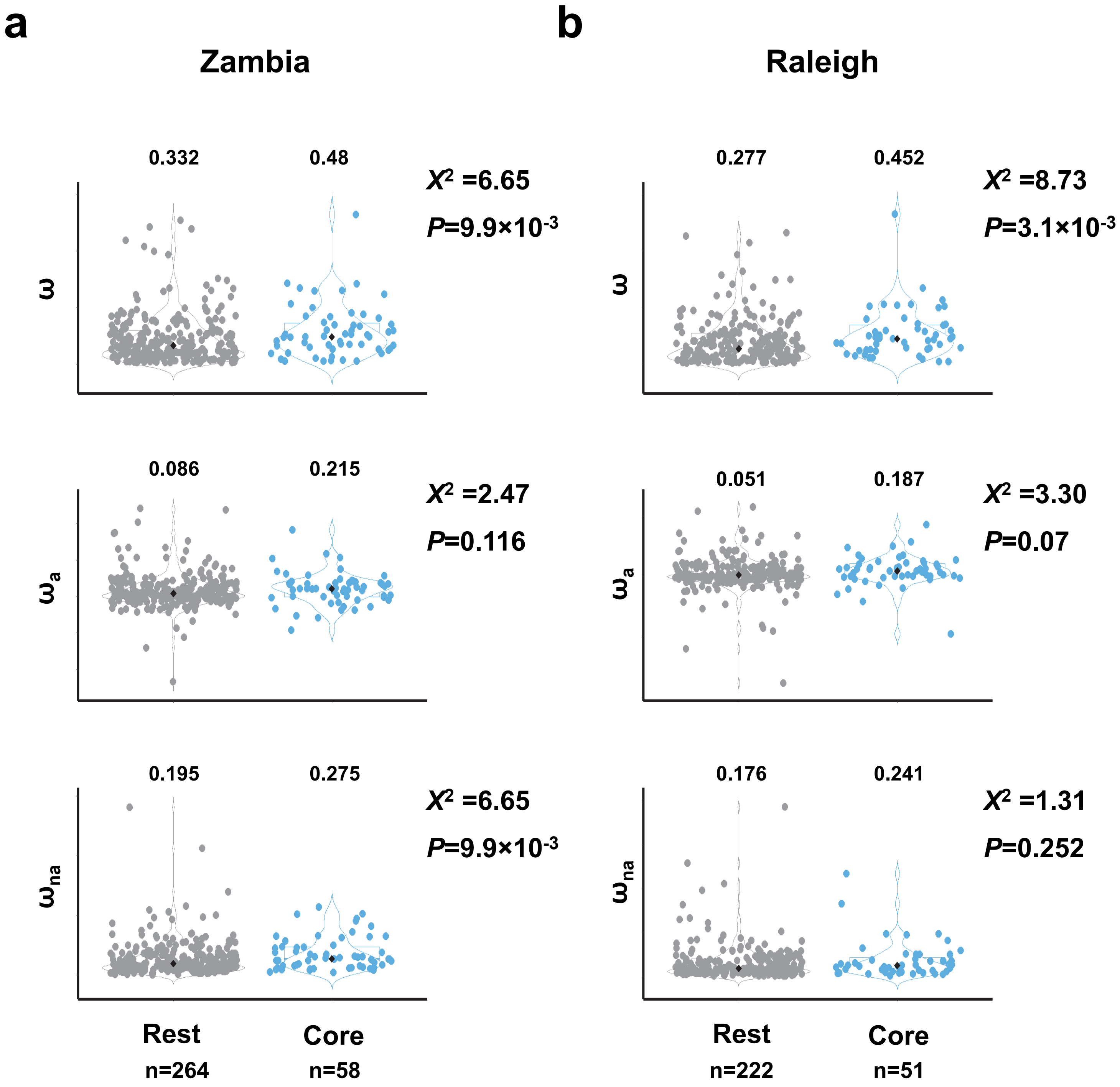

Figure S7. Rate of evolution of core subnetwork Sfp genes vs other Sfp genes.

a The ratio of nonsynonymous to synonymous substitutions (ω), plus the adaptive (ω_a) and non-adaptive (ω_{na}) rates of evolution, between *D. melanogaster* and *D. simulans* as estimated using data from a *D. melanogaster* Zambia population. **b** Same estimates using data from a Raleigh population of *D. melanogaster*. Violin and box plots are shown in the foreground and background, respectively. Boxes represent the interquartile range (IQR) around the median (black diamond) and whiskers extend to 1.5 times the IQR. The median value is shown on top. The *p*-value associated with the Wilcoxon rank sum test with continuity correction is provided on the right of each contrast.

Supporting Tables

• Table S1. Test for an even representation of Sfp genes across age classes.

• Table S2. Enrichment patterns for GO:Biological Process terms of Sfp genes from different age classes according to STRING.

• Table S3. Statistical significance associated with the post hoc tests for differences in the number of PPIs among Sfps encoded by genes that belong to different age classes

• Table S4. Statistical significance associated with the post hoc tests for differences in tissue-specificity expression (τ) index values across Sfp genes from different age classes

• Table S5. Test for an even representation of Sfp genes with maximum expression in male accessory glands across age classes.

• Table S6. Different sequence evolution metrics across different age classes of *Sfp* genes in two populations of *D. melanogaster.*

• Table S7. Statistical significance associated with the post hoc tests for differences in rates of evolution among different age classes of *Sfp* genes.

• Table S8. Test for an even representation of Sfps with reproductive roles across subnetworks.

• Table S9. Test for an even representation of Sfps with reproductive roles across subnetworks.

• Table S10. Test for differences in several metrics related to the rate of evolution among *Sfp* genes part or not of the core subnetwork.

Table S1. Test for an even representation of Sfp genes across age classes

	All Sfp Genes (n=357)			*Consensus Sfp Gene Set (n=228)*			*All Sfp Genes (n=357) — All Paralogs Omitted*			*All Sfp Genes (n=357) — A Limited Fraction Of Paralogs Considered †*		
**Age Class**	**n**	**Standardized Residuals**	P_{adj} *	**n**	**Standardized Residuals**	P_{adj} *	**n**	**Standardized Residuals**	P_{adj} *	**n**	**Standardized Residuals**	P_{adj} *
A	220	-13.89	<1.00E-06	125	-14.23	<1.00E-06	114	-11.96	<1.00E-06	185	-14.55	<1.00E-06
B	22	0.91	0.362	17	1.6	0.109	9	-0.35	0.727	22	1.52	0.053
C	28	4.67	3.80E-06	22	5.22	2.20E-07	17	-4.06	6.03E-05	26	4.79	2.24E-04
D	28	8.89	<1.00E-06	17	6.64	5.20E-11	20	9.08	<1.00E-06	26	8.91	<1.00E-06
E	58	14.67	<1.00E-06	47	15.56	<1.00E-06	37	12.96	<1.00E-06	55	15.05	<1.00E-06
		* $\chi2$ = 333.74, df = 4, P < 2.2E-16			* $\chi2$ = 333.71, df = 4, P < 2.2E-16			$\chi2$ = 278.83, df = 4, P < 2.2E-16			$\chi2$ = 350.03, df = 4, P < 2.2E-16	

A-E, gene age classes (see Fig. 1 for details).

Negative and positive standardized residuals are shown, denoting depletion and enrichment, respectively.

† Paralog groups in which all the paralogs belong to the same age class were represented by a single paralog.

* After multiple test correction (8).

Table S2. Enrichment patterns for GO:Biological Process terms of Sfp genes from different age classes according to STRING

Set	Five Age Classes	Gene Ontology ID	Description	Counts *	Strength ¦	P_{adj} ‡	Preradiation vs Radiation §	Gene Ontology ID	Description	Counts *	Strength ¦	P_{adj} ‡
All Sfp Genes (n=357)												
	A	GO:0000003	reproduction	118/1426	0.72	1.47E-52	preradiation	GO:0000003	reproduction	118/1426	0.72	1.47E-52
	A	GO:0005975	carbohydrate metabolic process	13/246	0.53	0.0327	preradiation	GO:0005975	carbohydrate metabolic process	13/246	0.53	0.0327
	A	GO:0006457	protein folding	11/141	0.69	0.0054	preradiation	GO:0006457	protein folding	11/141	0.69	0.0054
	A	GO:0006508	proteolysis	36/868	0.42	5.90E-05	preradiation	GO:0006508	proteolysis	36/868	0.42	5.90E-05
	A	GO:0007610	behavior	21/523	0.41	0.0201	preradiation	GO:0007610	behavior	21/523	0.41	0.0201
	A	GO:0009056	catabolic process	33/949	0.34	0.0048	preradiation	GO:0009056	catabolic process	33/949	0.34	0.0048
	A	GO:0018401	peptidyl-proline hydroxylation to 4-hydroxy-L-proline	6/27	1.15	0.0028	preradiation	GO:0018401	peptidyl-proline hydroxylation to 4-hydroxy-L-proline	6/27	1.15	0.0028
	A	GO:0019953	sexual reproduction	113/1143	0.8	7.61E-57	preradiation	GO:0019953	sexual reproduction	113/1143	0.8	7.61E-57
	A	GO:0043171	peptide catabolic process	5/29	1.04	0.0274	preradiation	GO:0043171	peptide catabolic process	5/29	1.04	0.0274
	A	GO:0046692	sperm competition	10/21	1.48	2.44E-08	preradiation	GO:0046692	sperm competition	10/21	1.48	2.44E-08
	A	GO:0051707	response to others organism	18/397	0.46	0.0164	preradiation	GO:0051707	response to others organism	18/397	0.46	0.0164
	A	GO:0052548	regulation of endopeptidase activity	16/132	0.89	6.45E-07	preradiation	GO:0052548	regulation of endopeptidase activity	16/132	0.89	6.45E-07
	A	GO:1901136	carbohydrate derivative catabolic process	9/74	0.89	0.0015	preradiation	GO:1901136	carbohydrate derivative catabolic process	9/74	0.89	0.0015
	A	GO:1901565	organonitrogen compound catabolic process	68/2785	0.19	0.0164	preradiation	GO:1901565	organonitrogen compound catabolic process	68/2785	0.19	0.0164
	B	GO:0019953	sexual reproduction	16/1143	0.95	1.5E-09	radiation	GO:0019953	Sexual reproduction	86/1143	0.89	1.06E-53
	C	GO:0019953	sexual reproduction	16/1143	0.84	3.75E-07	radiation	GO:0018991	oviposition	4/10	1.62	0.002
	C	GO:0007320	insemination	3/24	1.79	0.0481						
	D	GO:0019953	sexual reproduction	15/1143	0.82	5.15E-06						
	E	GO:0019953	sexual reproduction	40/1143	0.93	4.52E-26						
	E	GO:0045924	regulation of female receptivity	5/37	1.51	0.00048						
Consensus Sfp Gene Set (n=228)												
	A	GO:0000003	reproduction	93/1426	0.87	1.94E-60	preradiation	GO:0000003	reproduction	93/1426	0.87	1.94E-60
	A	GO:0006508	proteolysis	23/868	0.47	0.00075	preradiation	GO:0006508	proteolysis	23/868	0.47	0.00075
	A	GO:0007610	behavior	16/523	0.54	0.0048	preradiation	GO:0007610	behavior	16/523	0.54	0.0048
	A	GO:0009056	catabolic process	23/946	0.44	0.0027	preradiation	GO:0009056	catabolic process	23/946	0.44	0.0027
	A	GO:0019953	sexual reproduction	92/1143	0.96	5.58E-67	preradiation	GO:0019953	sexual reproduction	92/1143	0.96	5.58E-67
	A	GO:0043171	peptide catabolic process	4/29	1.19	0.0427	preradiation	GO:0043171	peptide catabolic process	4/29	1.19	0.0427

Table S2. Enrichment patterns for GO:Biological Process terms of Sfp genes from different age classes according to STRING

Set	Five Age Classes	Gene Ontology ID	Description	Counts *	Strength ¦	P_{adj} ‡	Preradiation vs Radiation §	Gene Ontology ID	Description	Counts *	Strength ¦	P_{adj} ‡
	A	GO:0046662	regulation of egg-laying behavior	3/10	1.53	0.0409	preradiation	GO:0046662	regulation of egg-laying behavior	3/10	1.53	0.0409
	A	GO:0046692	sperm competition	10/21	1.73	1.75E-10	preradiation	GO:0046692	sperm competition	10/21	1.73	1.75E-10
	A	GO:0052548	regulation of endopeptidase activity	13/132	1.04	2.09E-07	preradiation	GO:0052548	regulation of endopeptidase activity	13/132	1.04	2.09E-07
	B	GO:0019953	sexual reproduction	16/1143	1.06	5.12E-13	radiation	GO:0019953	sexual reproduction	82/1143	0.99	1.14E-64
	C	GO:0019953	sexual reproduction	16/1143	0.95	1.50E-09	radiation	GO:0018991	oviposition	4/10	1.74	0.00073
	C	GO:0007320	insemination	3/24	1.9	0.0228						
	D	GO:0019953	sexual reproduction	13/1143	0.97	9.89E-08						
	E	GO:0007617	mating behavior	7/139	1.17	0.0003						
All Sfp Genes (n=357) — All Paralogs Omitted												
	A	GO:0019953	Sexual reproduction	57/1143	0.79	3.21E-27	preradiation	GO:0019953	Sexual reproduction	57/1143	0.79	3.21E-27
	A	GO:0000003	Reproduction	58/1426	0.7	1.21E-23	preradiation	GO:0000003	Reproduction	58/1426	0.7	1.21E-23
	A	GO:0051707	Response to other organism	15/397	0.66	0.0027	preradiation	GO:0051707	Response to other organisms	15/397	0.66	0.0027
	A	GO:0006952	Defense response	13/372	0.63	0.0143	preradiation	GO:0006952	Defense response	13/372	0.63	0.0143
	A	GO:0006457	Protein folding	8/141	0.84	0.0247	preradiation	GO:0006457	Protein folding	8/141	0.84	0.0247
	A	GO:0009617	Response to bacterium	10/233	0.72	0.0247	preradiation	GO:0009617	Response to bacterium	10/233	0.72	0.0247
	A	GO:0042742	Defense response to bacterium	9/205	0.73	0.0427	preradiation	GO:0042742	Defense response to bacterium	9/205	0.73	0.0427
	C	GO:0019953	Sexual reproduction	9/1143	0.81	0.0161	radiation	GO:0019953	Sexual reproduction	46/1143	0.84	1.8E-24
	C	GO:0042628	Mating plug formation	2/4	2.61	0.0498	radiation	GO:0045924	Regulation of female receptivity	7/37	1.51	1.36E-05
	E	GO:0019953	Sexual reproduction	24/1143	0.9	9.1E-14	radiation	GO:0007617	Mating behavior	10/39	1.09	2.65E-05
	E	GO:0045924	Regulation of female receptivity	4/37	1.61	0.0092	radiation	GO:0045434	Negative regulation of female receptivity, post-mating	4/16	1.63	0.0049
							radiation	GO:0042628	Mating plug formation	3/4	2.11	0.0059
							radiation	GO:0007320	Insemination	4/24	1.45	0.0128
							radiation	GO:0018991	Oviposition	3/10	1.71	0.0249

A-E, gene age classes (see Fig. 1 for details).

* Number of proteins in the network / Number (network+background) of proteins with the same GO term.

¦ Measure of enrichment calculated as the log10 ratio of the observed number of proteins annotated with a term in the network to the expected number of proteins with the same term in a random network of the same size.

‡ *After* multiple test correction (Benjamini and Holberg 1995).

§ Pre- and radiation categories correspond to class A and all other classes combined, respectively.

Table S3. Statistical significance associated with the post hoc tests for differences in the number of PPIs among Sfps encoded by genes that belong to different age classes

Contrast	Age Class 1	Age Class 2			
All Interactions		A	B	C	D
	B	0.8			
	C	0.91	0.85		
	D	0.19	0.19	0.19	
	E	0.8	0.91	0.91	0.19
Only Interactions With Other Sfps					
	B	0.075			
	C	0.04	0.939		
	D	0.876	0.162	0.095	
	E	0.095	0.939	0.716	0.407
Only Interactions With Non-Sfps					
	B	0.461			
	C	0.082	0.782		
	D	0.284	0.962	1	
	E	0.225	0.962	0.431	0.782

A-E, gene age classes (see Fig. 1 for details).

Pairwise tests were done using Wilcoxon rank-sum tests with continuity correction.

P_{adj} after multiple test correction are shown (8).

Table S4. Statistical significance associated with the post hoc tests for differences in tissue-specificity expression (τ) index values across Sfp genes from different age classes

Set	Age Class 1	Age Class 2			
All Sfp Genes (n=357)		A	B	C	D
* $\chi 2$ = 38.685, df = 1, P = 8.09E-08	B	1.40E-03			
	C	1.80E-03	0.545		
	D	1.40E-03	0.545	0.545	
	E	5.60E-03	1	0.545	0.172
Consensus Sfp Gene Set (n=228)		A	B	C	D
* $\chi 2$ = 23.17, df = 1, P = 1.17E-04	B	0.0167			
	C	0.0885	0.2377		
	D	0.2087	0.1558	0.2377	
	E	0.0012	0.3485	0.8914	0.1629

A-E, gene age classes (see Fig. 1 for details).
Pairwise tests were done using Wilcoxon rank-sum tests with continuity correction. P_{adj} after multiple test correction are shown (8).
* According to the Kruskal-Wallis rank-sum test with continuity correction.

Table S5. Test for an even representation of Sfp genes with maximum expression in male accessory glands across age classes

	All Sfp Genes (n=357)			*Consensus Sfp Gene Set (n=228)*			*All Sfp Genes (n=357) — All Paralogs Omitted*			*All Sfp Genes (n=357) — A Limited Fraction Of Paralogs Considered †*		
**Age Class**	**n**	**Standardized Residuals**	P_{adj} *	**n**	**Standardized Residuals**	P_{adj} *	**n**	**Standardized Residuals**	P_{adj} *	**n**	**Standardized Residuals**	P_{adj} *
A	120	-15.47	<1.00E-06	84	-15.28	<1.00E-06	59	-13.57	<1.00E-06	105	-15.85	<1.00E-06
B	17	1.57	0.117	16	2.39	0.167	7	0.18	0.86	17	1.94	0.053
C	19	4.08	5.56E-05	18	5.01	6.70E-07	11	3.25	1.40E-03	17	3.75	2.24E-04
D	22	9.12	<1.00E-06	13	5.76	1.38E-08	17	10.03	<1.00E-06	22	9.7	<1.00E-06
E	52	17.4	<1.00E-06	45	17.52	<1.00E-06	34	15.6	<1.00E-06	50	17.59	<1.00E-06
	$\chi 2$ = 425.41, df = 4, $P < 2.2E-16$			$\chi 2$ = 390.84, df = 4, $P < 2.2E-16$			$\chi 2$ = 369.53, df = 4, $P < 2.2E-16$			$\chi 2$ =442.91, df = 4, $P < 2.2E-16$		

A-E, gene age classes (see Fig. 1 for details).

Negative and positive standardized residuals are shown, denoting depletion and enrichment, respectively.

† Paralog groups in which all the paralogs were expressed in male accessory glands and belong to the same age class were represented by only one paralog.

* After multiple test correction (8).

Table S6. Different sequence evolution metrics across different age classes of *Sfp* genes in two populations of *D. melanogaster*

All Sfp Genes (N=357)

Population	Metric	Five Age Classes: Age Class	Count	Median	Preradiation vs Radiation ‡: Age Class	Count	Median
ZI	**ω**	A	201	0.236	A	201	0.236
		B	19	0.483	B+C+D+E	120	0.577
		C	26	0.521			
		D	24	0.615	* χ^2 = 63.861, df = 1, P = 1.335e-15		
		E	51	0.665			
		* χ^2 = 67.497, df = 4, P = 7.658e-14					
	ωa						
		A	201	0.04	A	201	0.04
		B	19	0.227	B+C+D+E	120	0.253
		C	26	0.247			
		D	24	0.264	* χ^2 = 19.209, df = 1, P = 1.171e-05		
		E	51	0.316			
		* χ^2 = 19.645, df = 4, P = 5.87e-04					
	ωna						
		A	201	0.173	A	201	0.173
		B	19	0.197	B+C+D+E	120	0.312
		C	26	0.358			
		D	24	0.258	* χ^2 = 32.353, df = 1, P = 1.285e-08		
		E	51	0.395			
		* χ^2 = 39.101, df = 4, P = 6.64e-08					
RAL	ω						
		A	173	0.233	A	173	0.233
		B	19	0.446	B+C+D+E	100	0.565

Consensus Sfp Gene Set (N=228)

Population	Metric	Five Age Classes: Age Class	Count	Median	Preradiation vs Radiation ‡: Age Class	Count	Median
ZI	**ω**	A	116	0.264	A	116	0.264
		B	14	0.522	B+C+D+E	92	0.594
		C	20	0.561			
		D	16	0.713	* χ^2 = 36.847, df = 1, P = 1.278e-09		
		E	42	0.643			
		* χ^2 = 38.685, df = 4, P = 8.091e-08					
	ωa						
		A	116	0.0576	A	201	0.04
		B	14	0.252	B+C+D+E	120	0.253
		C	20	0.202			
		D	16	0.25	* χ^2 =10.507, df = 1, P = 0.001189		
		E	42	0.292			
		* χ^2 = 11.5, df = 4, P = 0.0215					
	ωna						
		A	116	0.185	A	116	0.0576
		B	14	0.221	B+C+D+E	92	0.254
		C	20	0.338			
		D	16	0.32	* χ^2 = 21.996, df = 1, P = 2.732e-06		
		E	42	0.38			
		* χ^2 = 26.649, df = 4, P= 2.341e-05					
RAL	ω						
		A	104	0.247	A	104	0.247
		B	14	0.449	B+C+D+E	76	0.58

All Sfp Genes (n=357) — All Paralogs Omitted

Population	Metric	Five Age Classes: Age Class	Count	Median	Preradiation vs Radiation ‡: Age Class	Count	Median
ZI	**ω**	A	100	0.195	A	100	0.195
		B	6	0.505	B+C+D+E	68	0.59
		C	15	0.603			
		D	17	0.604	* χ^2 = 43.541, df = 1, p-value = 4.152e-11		
		E	30	0.59			
		* χ^2 = 45.735, df = 4, P = 2.796e-09					
	ωa						
		A	100	0.0335	A	100	0.0335
		B	6	0.28	B+C+D+E	68	0.253
		C	15	0.252			
		D	17	0.255	* χ^2 = 10.907, df = 1, p-value = 9.582e-04		
		E	30	0.243			
		* χ^2 = 12.213, df = 4, p-value = 0.01584					
	ωna						
		A	100	0.16	A	100	0.16
		B	6	0.18	B+C+D+E	68	0.349
		C	15	0.502			
		D	17	0.332	* χ^2 = 30.784, df = 1, p-value = 2.884e-08		
		E	30	0.36			
		* χ^2 = 34.326, df = 4, p-value = 6.389e-07					
RAL	ω						
		A	83	0.152	A	83	0.152
		B	6	0.473	B+C+D+E	55	0.564

Table S6. Different sequence evolution metrics across different age classes of *Sfp* genes in two populations of *D. melanogaster*

All Sfp Genes (N=357)							
**Population**	**Metric**	**Five Age Classes**			**Preradiation vs Radiation ‡**		
		C	20	0.534			
		D	20	0.61	* χ^2 = 55.423, df = 1, *P* = 9.719e-14		
		E	41	0.672			
		* χ^2 = 57.347, df = 4, *P* = 1.046e-11					
	ωa						
		A	173	0.0139	A	173	0.0139
		B	19	0.237	B+C+D+E	100	0.275
		C	20	0.295			
		D	20	0.36	* χ^2 = 19.848, df = 1, *P* = 8.386e-06		
		E	41	0.282			
		* χ^2 = 21.091, df = 4, *P* = 3.04e-04					
	ωna						
		A	173	0.155	A	173	0.155
		B	19	0.18	B+C+D+E	100	0.27
		C	20	0.288			
		D	20	0.204	* χ^2 = 14.777, df = 1, *P* = 0.000121		
		E	41	0.455			
		* χ^2 = 20.391, df = 4, *P* = 4.18e-4					

Consensus Sfp Gene Set (N=228)							
**Population**	**Metric**	**Five Age Classes**			**Preradiation vs Radiation ‡**		
		C	15	0.578			
		D	13	0.66	* χ^2 = 36.011, df = 1, *P* = 1.962e-09		
		E	34	0.679			
		* χ^2 =37.421, df = 4, *P* = 1.475e-07					
	ωa						
		A	104	0.0488	A	104	0.0488
		B	14	0.255	B+C+D+E	76	0.288
		C	15	0.294			
		D	13	0.383	* χ^2 =11.601, df = 1, *P* = 0.000659		
		E	34	0.328			
		* χ^2 = 12.808, df = 4, *P* = 0.01225					
	ωna						
		A	104	0.15	A	104	0.15
		B	14	0.195	B+C+D+E	76	0.274
		C	15	0.259			
		D	13	0.277	* χ^2 = 11.355, df = 1, *P* = 0.0007523		
		E	34	0.406			
		* χ^2 = 13.431, df = 4, *P* = 0.00935					

All Sfp Genes (n=357) – All Paralogs Omitted							
**Population**	**Metric**	**Five Age Classes**			**Preradiation vs Radiation ‡**		
		C	11	0.553			
		D	14	0.571	* χ^2 = 39.079, df = 1, p-value = 4.07e-10		
		E	24	0.689			
		* χ^2 = 40.766, df = 4, p-value = 3.005e-08					
	ωa						
		A	83	-0.002	A	83	-0.002
		B	6	0.124	B+C+D+E	55	0.196
		C	11	0.296			
		D	14	0.29	* χ^2 = 8.8353, df = 1, p-value = 0.002955		
		E	24	0.109			
		* χ^2 = 10.973, df = 4, p-value = 0.02687					
	ωna						
		A	83	0.135	A	83	0.135
		B	6	0.196	B+C+D+E	55	0.382
		C	11	0.389			
		D	14	0.218	* χ^2 = 22.305, df = 1, p-value = 2.326e-06		
		E	24	0.492			
		* χ^2 = 26.719, df = 4, p-value = 2.265e-05					

ZI, Zambia; RAL, Raleigh. A-E, gene age classes (see Fig. 1 for details).

* According to the Kruskal-Wallis (>2 classes) or the Wilcoxon (2 classes) rank-sum test with continuity correction.

‡ Pre- and radiation categories correspond to class A and all other classes combined, respectively.

Table S7. Statistical significance associated with the post hoc tests for differences in rates of evolution among different age classes of *Sfp* genes

Population	Metric	***All Sfp Genes (n=357)***					Metric	***Consensus Sfp Gene Set (n=228)***				
ZI	ω	Age Class	A	B	C	D	ω	Age Class	A	B	C	D
		B	0.0104	-	-	-		B	0.04363	-	-	-
		C	1.10E-05	0.14496	-	-		C	0.00072	0.38463	-	-
		D	0.00074	0.27843	0.70738	-		D	0.02947	0.44368	0.9624	-
		E	4.60E-10	0.01468	0.32541	0.32605		E	2.80E-06	0.11947	0.40231	0.78708
	ω_a		A	B	C	D	ω_a		A	B	C	D
		B	0.0608	-	-	-		B	0.2	-	-	-
		C	0.1353	1	-	-		C	0.6	0.73	-	-
		D	0.1353	0.8852	1	-		D	0.6	0.98	0.98	-
		E	0.0035	0.8852	0.8852	1		E	0.03	0.98	0.6	0.98
	ω_{na}		A	B	C	D	ω_{na}		A	B	C	D
		B	0.3427	-	-	-		B	0.4484	-	-	-
		C	0.0004	0.0435	-	-		C	0.0043	0.106	-	-
		D	0.0554	0.4475	0.2645	-		D	0.106	0.4484	0.4484	-
		E	3.20E-06	0.0275	0.7589	0.1565		E	0.0002	0.0641	0.7595	0.4484
RAL	ω		A	B	C	D	ω		A	B	C	D
		B	0.00264	-	-	-		B	0.0206	-	-	-
		C	0.00019	0.32278	-	-		C	0.0022	0.3353	-	-
		D	0.00109	0.43468	0.98933	-		D	0.0105	0.465	0.9278	-
		E	3.90E-08	0.04053	0.43468	0.56349		E	1.50E-05	0.1034	0.6405	0.8746
	ω_a		A	B	C	D	ω_a		A	B	C	D
		B	0.04	-	-	-		B	0.35	-	-	-

Table S7. Statistical significance associated with the post hoc tests for differences in rates of evolution among different age classes of *Sfp* genes

**Population**	**Metric**	***All Sfp Genes (n=357)***					**Metric**	***Consensus Sfp Gene Set (n=228)***				
		C	0.027	0.52	-	-		C	0.11	0.53	-	-
		D	0.025	0.368	0.608	-		D	0.11	0.4	0.62	-
		E	0.04	0.608	0.8	0.539		E	0.11	0.56	0.99	0.62
	ω_{na}		A	B	C	D	ω_{na}		A	B	C	D
		B	0.51431	-	-	-		B	0.4232	-	-	-
		C	0.16703	0.51431	-	-		C	0.4232	0.724	-	-
		D	0.38168	0.70759	0.57354	-		D	0.4232	0.6877	0.9816	-
		E	0.00027	0.11994	0.29308	0.11994		E	0.0085	0.4232	0.4232	0.4232

ZI, Zambia; RAL, Raleigh. A-E, gene age classes (see Fig. 1 for details).

Pairwise tests were done using Wilcoxon rank-sum tests with continuity correction.

P_{adj} after multiple test correction are shown (8).

Table S8. Test for an even representation of Sfps with reproductive roles across subnetworks

	All Sfp Genes (n=357)			*Consensus Sfp Gene Set (n=228)*			*All Sfp Genes (n=357) — All Paralogs Omitted*			*All Sfp Genes (n=357) — A Limited Fraction Of Paralogs Considered †*		
**Subnetwork**	**Age Class**	**Sfp Number**	***P*(Excess)? ***	**Age Class**	**Sfp Number**	***P*(Excess)? ***	**Age Class**	**Sfp Number**	***P*(Excess)? ***	**Age Class**	**Sfp Number**	***P*(Excess)? ***
1	A	25	0.98	A	24	0.8521	A	6	0.9981	A	19	0.9803
	B	7	0.2652	B	7	0.6429	B	1	0.5104	B	6	0.4628
	C	8	0.3275	C	8	0.6758	C	3	0.5104	C	6	0.4761
	D	4	0.7791	D	3	0.8883	D	3	0.6165	D	4	0.8307
	E	20	0.0655	E	19	0.6429	E	9	0.0891	E	16	0.1588
	n	64		n	61		n	22		n	51	
2	A	13	0.0002	A	6	0.0324	A	13	0.0044	A	13	0.0015
	B	0	na	B	0	na	B	0	na	B	0	na
	C	0	na	C	0	na	C	0	na	C	0	na
	D	0	na	D	0	na	D	0	na	D	0	na
	E	0	na	E	0	na	E	0	na	E	0	na
	n	13		n	6		n	13		n	13	
3	A	1	na	A	1	na	A	0	na	A	1	na
	B	0	na	B	0	na	B	0	na	B	0	na
	C	1	na	C	1	na	C	1	na	C	1	na
	D	4	0.0014	D	3	0.0324	D	3	0.0315	D	4	0.0102
	E	1	na	E	1	na	E	0	na	E	1	na
	n	7		n	6		n	4		n	7	
4	A	4	0.3619	A						A	2	na
	B	1	na	B						B	1	na
	C	1	na	C						C	1	na
	D	0	na	D						D	0	na
	E	0	na	E						E	0	na
	n	6		n						n	4	
5	A	3	0.331	A			A	3	0.5104	A	3	0.4761
	B	0	na	B			B	0	na	B	0	na

Table S8. Test for an even representation of Sfps with reproductive roles across subnetworks

	All Sfp Genes (n=357)			Consensus Sfp Gene Set (n=228)			All Sfp Genes (n=357) — All Paralogs Omitted			All Sfp Genes (n=357) — A Limited Fraction Of Paralogs Considered †		
**Subnetwork**	**Age Class**	**Sfp Number**	***P*(Excess)? ***	**Age Class**	**Sfp Number**	***P*(Excess)? ***	**Age Class**	**Sfp Number**	***P*(Excess)? ***	**Age Class**	**Sfp Number**	***P*(Excess)? ***
	C	0	na	C			C	0	na	C	0	na
	D	0	na	D			D	0	na	D	0	na
	E	1	na	E			E	1	na	E	1	na
	n	4		n			n	4		n	4	
6	A	4	0.06746	A			A	4	0.2118	A	4	0.1588
	B	0	na	B			B	0	na	B	0	na
	C	0	na	C			C	0	na	C	0	na
	D	0	na	D			D	0	na	D	0	na
	E	0	na	E			E	0	na	E	0	na
	n	4		n			n	4		n	4	

A-E, gene age classes (see Fig. 1 for details).
Only subnetworks with four Sfps or more, and age classes with at least three Sfps, are considered.
† Paralog groups in which all the paralogs belong to the same age class were represented by a single paralog.
* Probability of getting the observed number, or higher, of Sfps in 100,000 Monte Carlo simulations involving reshuffling without replacement. The resulting *p*-values were corrected for multiple tests (8).

Table S9. Test for an even representation of Sfps with reproductive roles across subnetworks

	Reproductive Role? ‡					
	All Sfp Genes (n=357)			***Consensus Sfp Gene Set (n=228)***		
**Subnetwork**	**Yes**	**No**	**P_{adj} ***	**Yes**	**No**	**P_{adj} ***
1 (Core)	6.241	-6.241	<1.00E-08	4.919	-4.919	<1.00E-08
2	-5.462	5.462	<1.00E-08	-6.418	6.418	<1.00E-08
3	0	0	1	-0.221	0.221	1
4	-3.997	3.997	<1.00E-08	na	na	na
5	-2.099	2.099	0.215	na	na	na
6	1.292	-1.292	1	na	na	na

Negative and positive standardized residuals are shown, denoting depletion and enrichment, respectively. Only subnetworks with four Sfps or more are considered.

* After multiple test correction (8).

‡ Reproductive role is defined as having associated at least one of the following GO terms: sexual reproduction, reproduction, sperm storage, sperm competition, regulation of female receptivity, mating behavior, and insemination.

Table S10. Test for differences in several metrics related to the rate of evolution among *Sfp* genes part or not of the core subnetwork

Set	Population	Metric	Network Fraction	Count	Median	χ^2	*P* *
All Sfp Genes (n=357)	ZI	ω	Core	58	0.48	6.6538	0.009895
			Rest	264	0.332		
		ω_a	Core	58	0.215	2.4702	0.116
			Rest	264	0.0863		
		ω_{na}	Core	58	0.275	6.6538	0.009895
			Rest	264	0.195		
	RAL	ω	Core	51	0.452	8.7266	0.003136
			Rest	222	0.277		
		ω_a	Core	51	0.187	3.2955	0.06947
			Rest	222	0.0513		
		ω_{na}	Core	51	0.241	1.3103	0.2523
			Rest	222	0.176		
Consensus Sfp Gene Set (n=228)	ZI	ω	Core	55	0.493	2.339	0.1262
			Rest	153	0.36		
		ω_a	Core	55	0.214	0.7795	0.3773
			Rest	153	0.108		
		ω_{na}	Core	55	0.294	4.5599	0.03273
			Rest	153	0.206		
	RAL	ω	Core	48	0.464	3.9705	0.0463
			Rest	132	0.342		

Table S10. Test for differences in several metrics related to the rate of evolution among *Sfp* genes part or not of the core subnetwork

Set	Population	Metric	Network Fraction	Count	Median	χ^2	*P* *
		ω_a	Core	48	0.211	0.82043	0.3651
			Rest	132	0.0914		
		ω_{na}	Core	48	0.242	1.8371	0.1753
			Rest	132	0.184		

ZI, Zambia; RAL, Raleigh.

* According to the Wilcoxon rank-sum test with continuity correction.

Supporting Datasets

• Dataset S1 (separate file). Information associated for Sfp-encoding genes across different functional and evolutionary features and metrics.

SI References

1. B. Song *et al.*, Conserved noncoding sequences provide insights into regulatory sequence and loss of gene expression in maize. *Genome research* **31**, 1245-1257 (2021).

2. R. Kafri, A. Bar-Even, Y. Pilpel, Transcription control reprogramming in genetic backup circuits. *Nature genetics* **37**, 295-299 (2005).

3. Y. Hu *et al.*, An integrative approach to ortholog prediction for disease-focused and other functional studies. *BMC Bioinformatics* **12**, 357 (2011).

4. D. P. Leader, S. A. Krause, A. Pandit, S. A. Davies, J. A. T. Dow, FlyAtlas 2: a new version of the Drosophila melanogaster expression atlas with RNA-Seq, miRNA-Seq and sex-specific data. *Nucleic Acids Res* **46**, D809-D815 (2018).

5. P. M. O'Grady, R. DeSalle, Phylogeny of the Genus Drosophila. *Genetics* **209**, 1-25 (2018).

6. B. M. Wiegmann *et al.*, Episodic radiations in the fly tree of life. *Proc Natl Acad Sci U S A* **108**, 5690-5695 (2011).

7. C. Dong *et al.*, Subcellular Enrichment Patterns of New Genes in Drosophila Evolution. *Mol Biol Evol* **42** (2025).

8. Y. Benjamini, Y. Hochberg, Controlling the False Discovery Rate - a Practical and Powerful Approach to Multiple Testing. *Journal of the Royal Statistical Society Series B-Methodological* **57**, 289-300 (1995).

9. J. Hurtado, F. C. Almeida, S. A. Belliard, S. Revale, E. Hasson, Research gaps and new insights in the evolution of Drosophila seminal fluid proteins. *Insect Mol Biol* **31**, 139-158 (2022).

10. S. Wigby *et al.*, The Drosophila seminal proteome and its role in postcopulatory sexual selection. *Philos Trans R Soc Lond B Biol Sci* **375**, 20200072 (2020).
